# Stress-induced precocious aging in PD-patient iPSC-derived NSCs may underlie the pathophysiology of Parkinson’s disease

**DOI:** 10.1038/s41419-019-1313-y

**Published:** 2019-02-04

**Authors:** Liang Zhu, Chenxi Sun, Jie Ren, Guangming Wang, Rongjie Ma, Lixin Sun, Danjing Yang, Shane Gao, Ke Ning, Zhigang Wang, Xu Chen, Shengdi Chen, Hongwen Zhu, Zhengliang Gao, Jun Xu

**Affiliations:** 1 East Hospital, Tongji University School of Medicine, Shanghai, China; 2 Department of Neuroscience, Sheffield Institute for Translational Neuroscience (SITraN), University of Sheffield, Sheffield, UK; 3 Shanghai Myfun Medical Cosmetology Hospital, Shanghai, China; 4 Shanghai Eighth People’s Hospital Affiliated to Jiangsu University, Shanghai, China; 5 Department of Neurology and Institue of Neurology, Ruijin Hospital affiliated to Shanghai Jiaotong University School of Medicine, Shanghai, China; 6 Tianjin Hospital, Tianjin Academy of Integrative Medicine, Tianjin, China; 7 Shanghai Tenth People’s Hospital, Tongji University School of Medicine, Shanghai, China; 8 Advanced Institute of Translational Medicine, Tongji University School of Medicine, Shanghai, China

## Abstract

Parkinson’s disease (PD) is an aging-related degenerative disorder arisen from the loss of dopaminergic neurons in substantia nigra. Although many genetic mutations have been implicated to be genetically linked to PD, the low incidence of familial PD carried with mutations suggests that there must be other factors such as oxidative stress, mitochondrial dysfunction, accumulation of misfolded proteins, and enhanced inflammation, which are contributable to the pathophysiology of PD. The major efforts of current research have been devoted to unravel the toxic effect of multiple factors, which directly cause the degeneration of dopaminergic neurons in adulthood. Until recently, several studies have demonstrated that NSCs had compromised proliferation and differentiation capacity in PD animal models or PD patient-derived iPS models, suggesting that the pathology of PD may be rooted in some cellular aberrations at early developmental stage but the mechanism remains to be elusive. Based on the early-onset PD patient-specific iPSCs, we found that PD-patient iPSC-derived NSCs were more susceptible to stress and became functionally compromised by radiation or oxidative insults. We further unraveled that stress-induced SIRT1 downregulation leading to autophagic dysfunction, which were responsible for these deficits in PD-NSCs. Mechanistically, we demonstrated that stress-induced activation of p38 MAPK suppressed SIRT1 expression, which in turn augmented the acetylation of multiple ATG proteins of autophagic complex and eventually led to autophagic deficits. Our studies suggest that early developmental deficits may, at least partially, contribute to the pathology of PD and provide a new avenue for developing better therapeutic interventions to PD.

## Introduction

Parkinson’s disease (PD) is one of the most common neurodegenerative diseases, which is characterized by movement abnormalities of PD patients such as tremor, rigidity, bradykinesia, and gait dysfunction^1^. The main pathological feature of PD is the selective loss of dopamine (DA) neurons in substantia nigra pars compacta of midbrain accompanied by reduced dopaminergic innervation of striatum as well as accumulation of Lewy bodies, primarily composed of αα-Synuclein^2,3^. However, the etiology of PD largely remains unknown^4,5^. One of the reasons is probably due to the lack of human PD models, which is incompetent to well recapitulate the genetic background and the progression of the disease. With the advent of the induced pluripotent stem cells (iPSCs) technology^6,7^, it becomes realistic to generate iPSCs from fibroblasts of PD patients and subsequently differentiate into DA neurons and potentially create human cell-based PD disease models. Since then, a lot of familial and sporadic PD iPSC lines as well as their isogenic control lines by gene-editing, have been generated and provide insightful clues regarding the mechanism of the disease^8,9^.

It is worthy of notice that some studies reported that PD iPSCs manifested early developmental defects at the stage of neural stem cells (NSCs)^10^. Consistently, several studies in mouse models have proved that NSCs carrying the SNCA mutation showed reduced proliferation, impaired neurogenesis, and increased cell death^11–14^. NSCs from transgenic mice expressing mutant LRRK2 exhibited deficient proliferation and reduced newborn neurons^15,16^. It is likely that insults, which lead to the degeneration of DA neurons may root at the very beginning of CNS development.

In the current studies, we found that when treated with irradiation or oxidative stress, NSCs derived from early-onset PD patients showed precocious senescence as well as compromised proliferation and neuronal differentiation. We further proved that stress-induced downregulation of SIRT1, which subsequently led to the autophagic dysfunction, was essential for the aforementioned phenotypes. Moreover, the defects in PD-NSCs would affect the development of brain organoids. These findings provided compelling evidence to support the idea that early developmental deficits in PD may contribute to the decline of DA neuron pool in adulthood and the molecular mechanism outlined in this study might facilitate the development of novel diagnostic and therapeutic maneuvers for fighting PD.

## Results

### Generation and characterization of human iPSCs from adult human dermal fibroblasts of PD patients

We got skin tissues from two early-onset idiopathic PD patients and one healthy individual as control by skin biopsy. One of the patients was a 35-year-old male PD patient with the disease onset at the age of 28 while another 21-year-old male with onset at age 19. The healthy control was a 28-year-old male with no history of neurological disease.

Human fibroblasts were isolated and expanded and their identities were confirmed by TE-7 staining (Fig. S2a). First, we conducted genetic analyses of genomic DNA by using whole-exome sequencing. All the detailed results were listed in Supplementary Table 1. In order to confirm these point mutations revealed by whole-exome sequencing, we identified them by using PCR assays and DNA-sequencing analysis. A rare insertion mutation in phospholipase A2, group VI (PLA2G6), c.28dupA (p.T10fs), was identified in the sample from 35-year-old PD patient (PD-fibroblasts) (Fig. S1a). The insertion mutation c.28dupA (p.T10fs) in PLA2G6 could cause a frameshift. We also found a potentially deleterious splice-site mutation c.292-1G > A within Parkin co-regulated gene (PACRG) in PD-S-fibroblasts (Fig. S1b), which derived from the 21-year-old PD patient, leading to an abnormal splice-site variants. Also, in addition to the two identified mutations, we couldn’t exclude that there were other mutations as revealed by whole-exome sequencing in PD samples.

Then skin fibroblasts were reprogrammed with standard methods. We picked 10–20 human embryonic stem cell (hESC)-like colonies per individual for further expansion, totaling 50 iPSC lines. At least 2 iPSC lines per patient and 2 iPSC lines per healthy control were thoroughly characterized and sustained long-term passaging (>20 passages) and then differentiated into neural stem cells for detailed analysis.

Both iPSCs from PD patients and from healthy control exhibited morphology similar to hESCs and were stained positively by alkaline phosphatase and typical hESC markers such as OCT4, NANOG, TRA1-60, TRA1-81, SSEA3, and SSEA4 (Fig. S2b, c). Reverse transcription-PCR (RT-PCR) analysis showed that iPSCs expressed many hESC-marker genes, such as *SOX2*, *OCT4*, *c-MYC*, *KLF4*, *NANOG*, *LIN28*, and *REX1* (Fig. S2d). Further analysis showed that iPSCs were hypomethylated at the OCT4 promoter regions compared to fibroblasts (Fig. S2e). These human iPSCs were able to differentiate into cells of three germ layers spontaneously in vitro, which were stained positively for βIII-tubulin (Tuj1, a marker of ectoderm), α-SMA (mesoderm), and AFP (endoderm) (Fig. S2f). Real-time quantitative PCR (Q-PCR) and RT-PCR confirmed that these differentiated cells expressed characteristic genes of three germ layers including SOX17, AFP, and FOXA (endoderm markers); SMA, BRACHYURY, and MSX1 (mesoderm markers); and GFAP, PAX6, and NCAM (ectoderm) (Fig. S2g, h). The teratoma contained tissues from all three germ layers including neural tissues (ectoderm), cartilage (mesoderm), and gut-like epithelial tissues (endoderm) (Fig. S2i). Taken together, all these iPSCs were very similar to hESCs in terms of cell morphology, pluripotency, gene markers, differentiation potency, and epigenetics.

### NSCs derived from PD patients manifest compromised proliferation and differentiation capacity and are susceptible to genotoxic stress

**Fig. 1:**
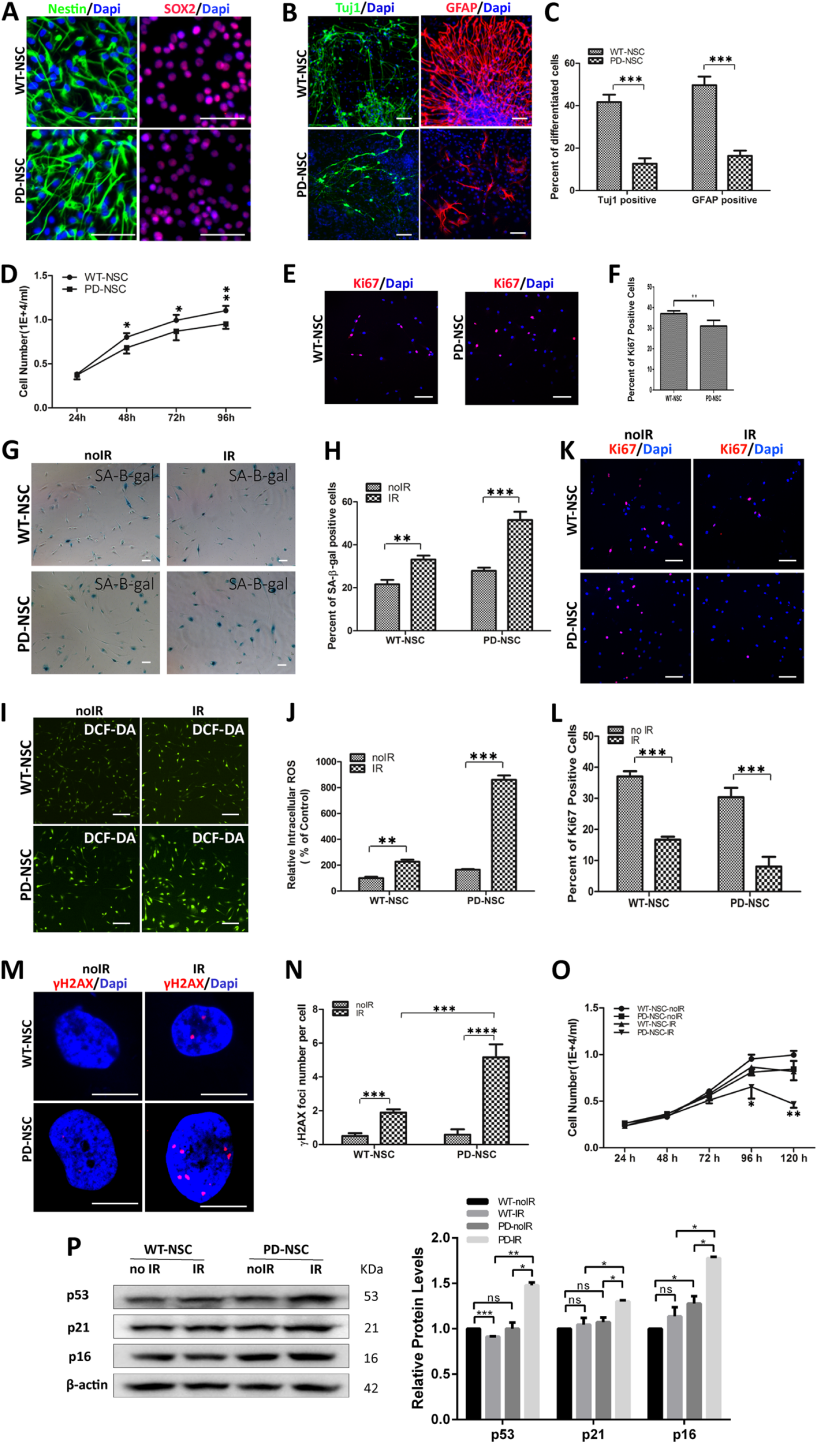
NSCs derived from patient-specific PD iPSCs manifested premature aging phenotypes and were sensitive to irradiation. **a** iPSC-derived neural stem cells were stained positively for NSC-specific markers Nestin and SOX2. Scale bar: 100 μm. **b**, **c** Neural differentiation capacity was severely impaired in PD iPSCs. **b** The representative images for spontaneous differentiation of PD- and WT-NSCs toward neurons and astrocytes as determined by the neuronal marker (Tuj1) and the astrocyte marker (GFAP). **c** Statistic results showed the percentage of Tuj1 and GFAP cells out of all cells derived from WT- and PD iPSCs. Scale bar: 100 μm. **d**–**f** The proliferative capacity of PD-NSCs was compromised as revealed by CCK8 assays and Ki67 staining. **d** Cell growth curve was detected by CCK8 assays at 24, 48, 72, 96, and 120 h. **e** Proliferation capacity was determined by Ki67 staining. **f** Graph showed the percentage of Ki67 cells. Scale bar: 100 μm. **g**, **h** Cellular senescence was significantly aggravated by IR treatment as evaluated with the SA-β-gal assay. **g** Representative images from at least three independent experiments were presented. **h** The statistical results showed the percent of SA-β-gal-positive cells (%). Scale bar: 100 μm. **i**, **j** ROS level increased significantly in PD-NSC with IR treatment. **i** Cellular ROS was stained with DCF-DA and observed under a fluorescence microscope. **j** ROS level was detected by fluorescence microplate reader. Scale bar: 100 μm. **k**, **l** Proliferative capacity was further decreased in PD-NSCs after IR treatment as revealed by Ki67 staining. **k** Expression of Ki67 in cells with or without IR treatment. **l** Graph showed the ratio of Ki67-positive cells. Scale bar: 100 μm. **m**, **n** DNA damage accumulation increased dramatically in PD-NSCs by IR as revealed by γH2AX staining (red) at 48 h post IR treatment. **m** Formation of DNA double-strand breaks in NSCs post irradiation. **n** Graphical depiction indicated the number of γH_2_AX foci in NSCs. Scale bar: 10 μm. **o** Cell proliferation curve of WT-NSCs and PD-NSCs with or without IR. Cell numbers were analyzed at 24, 48, 72, 96, and 120 h. **p** The expression level of cell senescence markers p53, p21, and p16 was augmented in PD-NSCs with IR treatment, analyzed by western blotting and densitometry. NSCs were treated with 10 Gy of IR and incubated for the indicated periods. β-actin was used as the loading control. All the data were expressed as mean ± SD. *P < 0.05, **P < 0.01, ***P < 0.001, ns not statistically significant, Student’s *t*-test. All data were obtained from at least three independent experiments

As aforementioned that the pathology of PD may start as early as NSC stage^10^, we investigated the differences between NSCs derived from PD patients (PD-NSCs, 35-year-old male and PD-S-NSCs, 21-year-old male) and healthy individual (WT-NSCs, 28-year-old male). All these NSCs expressed typical NSC markers Nestin and SOX2 with high purity (over 95%, Fig. 1a). Upon withdraw of basic fibroblast growth factor (bFGF), we observed that WT-NSCs spontaneously differentiated into Tuj1-positive neurons (42%) and GFAP-positive astrocytes (50%), whereas only less than one-third of PD-NSCs started spontaneous differentiation (Fig. 1b, c). Meanwhile, the proliferative capacity of PD-NSCs also reduced slightly as evaluated by cell counting kit-8 (CCK8) assays and Ki67 staining compared with WT-NSCs (Fig. 1d–f).

We further examined whether PD-NSCs were more susceptible to genotoxic stress by exposure to ionizing radiation (IR, 10 Gy of X-ray). PD-NSCs were more prone to become senescent than WT-NSCs, when insulted by IR, as estimated by SA-β-gal staining (Fig. 1g, h). We also observed these senescent phenotypes in NSCs derived from 21-year-old early-onset PD patient (PD-S-NSCs) (Fig. S4). The reactive oxygen species (ROS) level of PD-NSCs increased dramatically 48 h after IR treatment (5.2 folds) compared with wild-type (WT) control (2.3 folds) (Fig. 1i, j). The number of actively dividing NSCs (Ki67-positive) decreased more rapidly in PD-NSCs than WT-NSCs (3.8 folds vs 2.2 folds, Fig. 1k, l), which was accompanied by far more DSB DNA damage (γH2AX-positive foci) accumulation in PD-NSCs (Fig. 1m, n). PD-NSCs demonstrated most compromised proliferative ability when treated by IR (Fig. 1o). Meanwhile, IR treatment elicited a significant elevated expression of p16, p21, and p53, which were key regulators of cell senescence, in PD-NSCs with little effect in WT-NSCs (Fig. 1p).

### SIRT1 plays an essential role in regulating the precocious aging of PD-NSCs induced by genotoxic stress

**Fig. 2:**
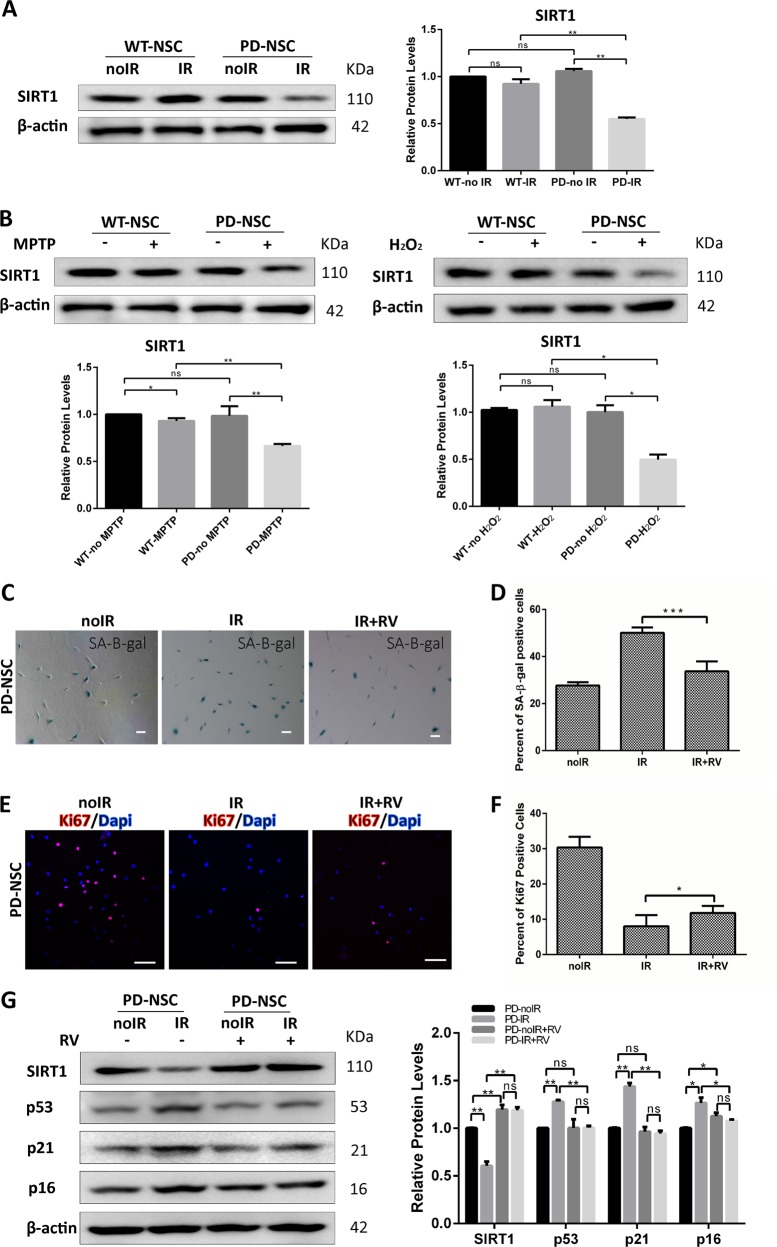
SIRT1 was downregulated in PD-NSCs by various stresses, which was attributable for the premature aging phenotypes. **a**, **b** SIRT1 expression in PD-NSCs was significantly reduced by various stresses including IR, MPTP, and oxidative stress treatment, analyzed by western blotting and densitometry. WT-NSCs and PD-NSCs were treated with 10 Gy of IR, 500 μM MPTP, and 100 μM H_2_O_2_, respectively, and then cultured for 48 h before western blot. **c**, **d** IR-induced cell senescence was largely attenuated by activation of SIRT1 in the presence of 3 μM resveratrol. **e**, **f** Resveratrol partially rescued the declined proliferative capacity (Ki67 staining) of PD-NSCs after IR treatment. **g** Induction of aging-related genes in PD-NSCs by IR treatment was prevented by resveratrol, analyzed by western blotting and densitometry. All data were obtained from at least three independent experiments; mean ± SD, *P < 0.05, **P < 0.01, ***P < 0.001, ns not statistically significant, Student’s *t*-test. Scale bar: 100 μm

SIRT1 is class III histone deacetylases, which can regulate cellular senescence^17–19^. Given that we sought to explore whether the premature aging of PD-NSCs was attributable to stress response of SIRT1. We found that the protein level of SIRT1 reduced markedly 48 h after IR treatment in PD-NSCs, whereas no significant changes detected in WT-NSCs (Fig. 2a). Similarly, SIRT1 was also downregulated by oxidative stress when treated with 1-Methyl-4-phenyl-1,2,3,6-tetrahydropyridine (MPTP) and H_2_O_2_ in PD-NSCs (Fig. 2b), suggesting that downregulation of SIRT1 was a general stress response specific for PD-NSCs. Also, as results showed in Fig. S6a, the reduced expression of SIRT1 was observed in PD-S-NSCs treated with oxidative stress. Cells with serial passaging (passage 20 and above) could resemble cellular aging^10^. We investigated the SIRT1 protein level in senile PD-S-NSCs at late passages. The results showed that SIRT1 expression in PD-S-NSCs at passage 25 was much lower than that in WT-NSCs (Fig. S6b).

We treated PD-NSCs with resveratrol, a well-known activator of SIRT1. Resveratrol partially rescued the senescence of PD-NSCs in response to IR and preserved more proliferative NSCs as revealed by SA-β-gal and Ki67 staining, respectively (Fig. 2c–f). The results also showed that resveratrol could resist the premature aging of PD-S-NSCs (Fig. S5). Consistently, activation of SIRT1 by resveratrol blocked the upregulation of p53, p21, and p16 in PD-NSCs after IR (Fig. 2g) and largely abolished the reduction of SIRT1 expression induced by IR (Fig. 2g).

**Fig. 3:**
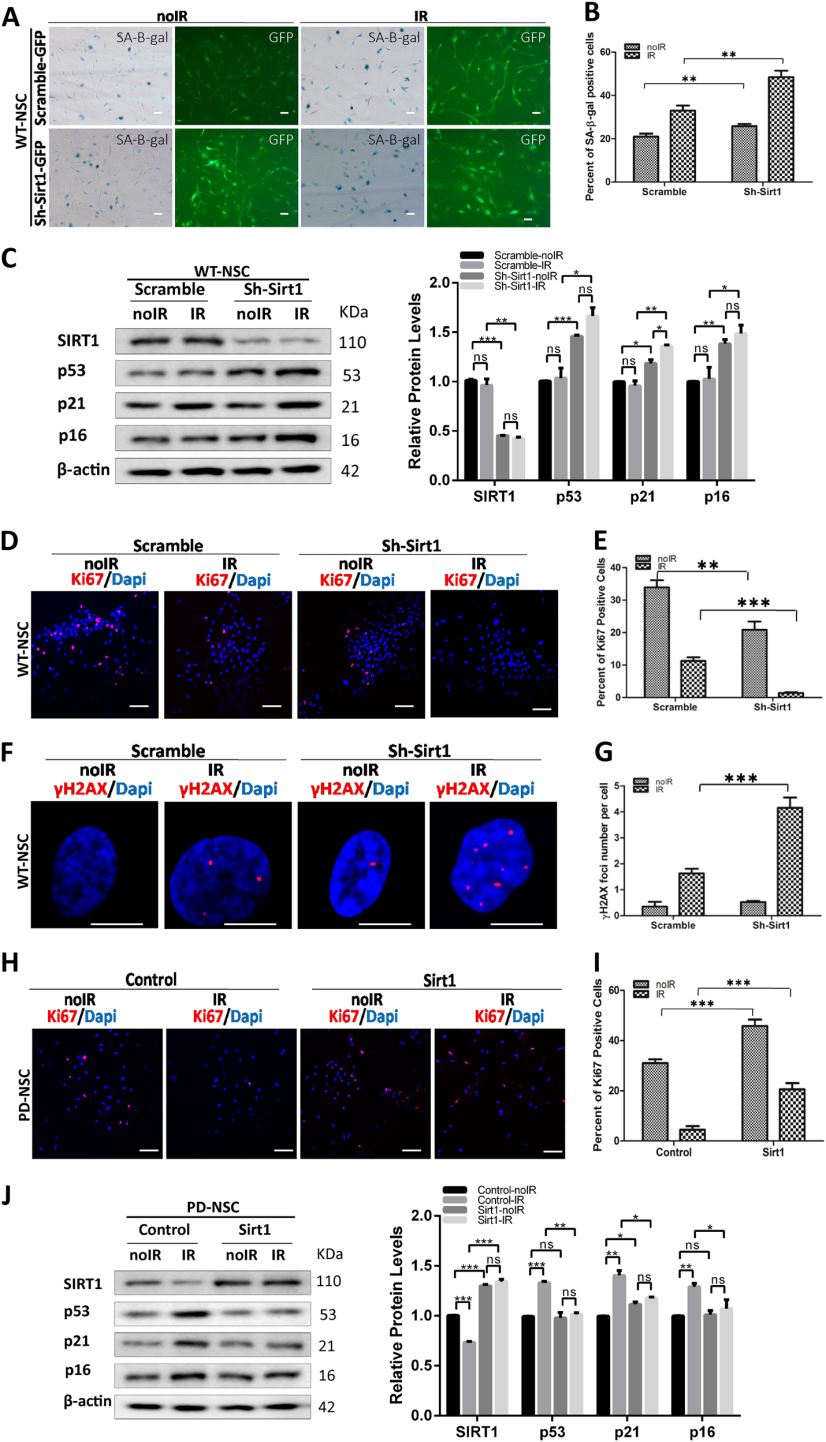
SIRT1 regulated the aging process of PD-NSCs after IR. **a**, **b** Cell senescence induced by IR was aggravated by downregulation of SIRT1 in WT-NSCs. WT-NSCs infected with SIRT1 shRNA were exposed to 10 Gy IR and incubated for 48 h. Scale bar: 100 μm. **c** Protein levels of aging-related genes in WT-NSCs with Sirt1 knockdown increased significantly, analyzed by western blotting and densitometry. **d**, **e** The expression of Ki67 decreased by knockdown of Sirt1 in WT-NSCs after IR treatment. Scale bar: 100 μm. **f**, **g** DNA damage accumulation represented by γH2AX foci enhanced dramatically in WT-NSCs with downregulated Sirt1 expression at 48 h post IR treatment. Scale bar: 10 μm. **h**, **i** Proliferative capacity as revealed by Ki67 staining was increased by overexpression of Sirt1 in PD-NSCs after IR treatment. SIRT1 expression in PD-NSCs was increased using a recombinant lentivirus encoding wild-type SIRT1, then cells were exposed to 10 Gy IR and incubated for 48 h. Scale bar: 100 μm. **j** Aging-related genes in PD-NSCs with Sirt1 overexpression further decreased, analyzed by western blotting and densitometry. All data were obtained from at least three independent experiments; mean ± SD, ***P < 0.001, **P < 0.01, *P < 0.05, ns not statistically significant, Student’s *t*-test

We sought to knockdown SIRT1 in WT-NSCs with a recombinant lentivirus encoding SIRT1 short hairpin RNA (shRNA) (Fig. 3c). Knockdown of SIRT1 only slightly made WT-NSCs more senescent while greatly aggravated the senescence of WT-NSCs upon IR treatment (Fig. 3a, b), accompanied by augmented expression of several aging-related genes such as *p53*, *p21*, and *p16* (Fig. 3c). Coincidentally, the proliferative NSCs were almost completely depleted by SIRT1 KD in WT-NSCs when challenged by IR (Fig. 3d, e). As expected, the DNA damage accumulation rocketed when insulted by IR in the absence of SIRT1 (Fig. 3f, g). These data suggested that SIRT1 downregulation was sufficient to predispose NSCs to become prematurely aged in response to stress. We attempt to rescue these aging phenotypes in PD-NSCs by overexpression of SIRT1. Indeed, the ectopic expression of SIRT1 led to a significant increase in Ki67 activity in PD-NSCs and the depletion of proliferative cells by IR was markedly inhibited (Fig. 3h, i). IR-induced upregulation of a spectrum of aging genes (*p53*, *p21*, and *p16*) was also nearly completely blocked by SIRT1 overexpression (Fig. 3j). Taken together, these data proved that SIRT1 played a pivotal role in maintaining the juvenescence and stemness of NSCs.

### SIRT1 expression level is modulated by p-p38 activities in PD-NSCs in response to IR

**Fig. 4:**
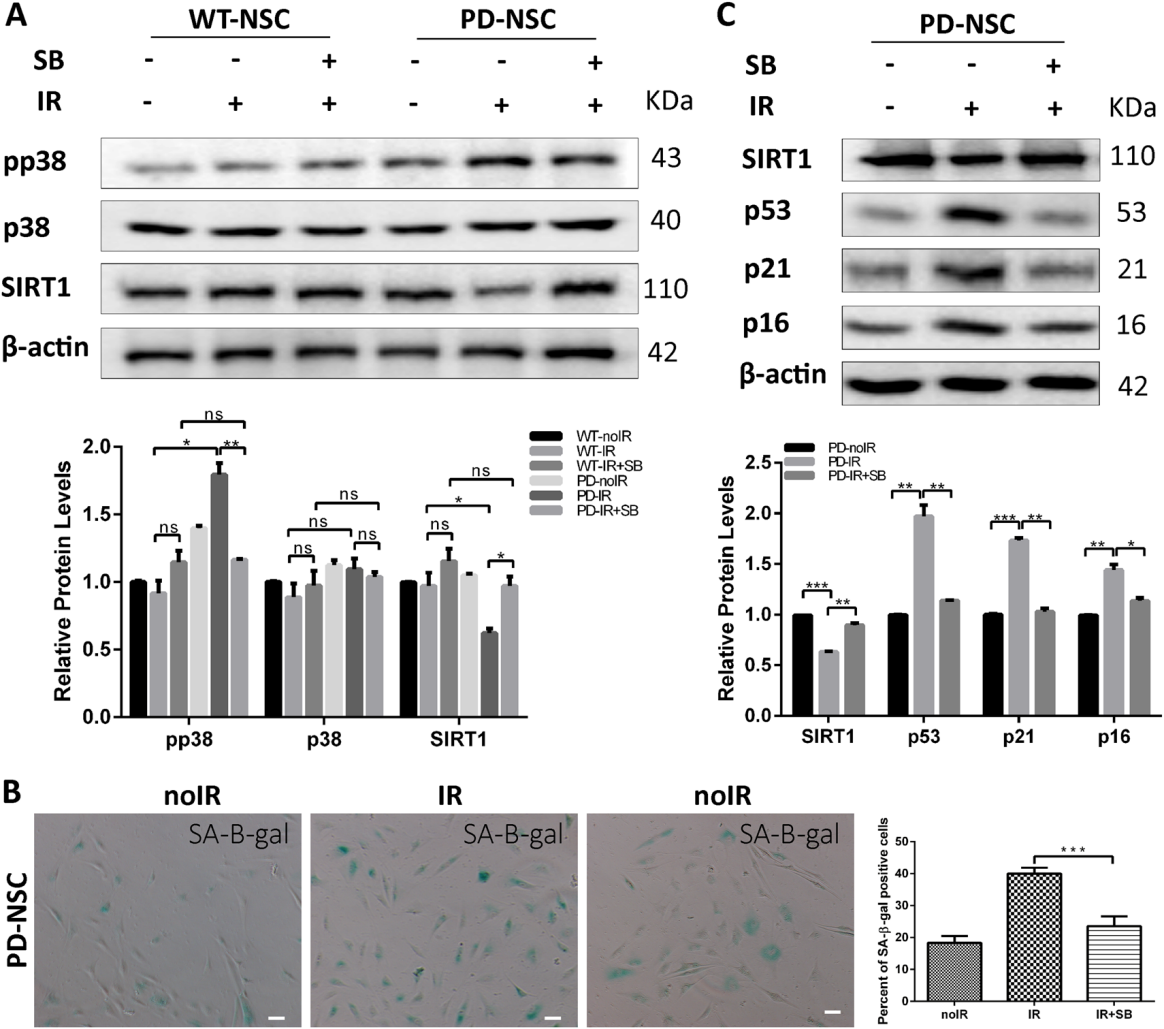
p-p38 could modulate SIRT1 expression in PD-NSCs after IR. **a** Gene expression of pP38 and SIRT1 was detected by western blot in WT-NSCs and PD-NSCs in the presence of p38 kinase-specific inhibitor, SB203580, analyzed by western blotting and densitometry. NSCs were irradiated 10 Gy and then incubated for 48 h in absence or presence of 20 μM SB203580. **b** IR-induced cell senescence was largely alleviated by suppression of pP38 in the presence of 20 μM SB203580. **c** The expression of aging-related genes *p16*, *p21*, and *p53* was further decreased in PD-NSCs in the presence of 20 μM SB203580, analyzed by western blotting and densitometry. Data represent the mean ± SD of three independent experiments. Statistical analysis was performed by Student’s *t*-test, ***P < 0.001, **P < 0.01, *P < 0.05, ns not statistically significant. All data were obtained from at least three independent experiments. Scale bar = 100 μm

Some studies reported that p38 kinase could modulate the degradation of SIRT1^20^ and be involved in the onset of cellular senescence^21–23^. Given that we investigated how p38 kinase modulated the expression of SIRT1. As shown in Fig. 4a, IR triggered a significant increase of the phosphorylation of p38 in PD-NSCs. However, the p-p38 level in WT-NSCs remained almost unaltered. Administration of p38 kinase-specific inhibitor, SB203580, almost completely blocked IR-induced downregulation of SIRT1 in PD-NSCs (Fig. 4a). Subsequently, by inhibition of p38 activities, the IR-triggered senescence was largely prevented (Fig. 4b) and the upregulation of aging-related genes, such as *p53*, *p21*, and *p16* were significantly suppressed (Fig. 4c). Our data collectively suggested that p-p38 acted as an upstream regulator of SIRT1 and participated in the aging of PD-NSCs.

### Autophagic dysfunction is responsible for the stress-induced premature aging of PD-NSCs

**Fig. 5:**
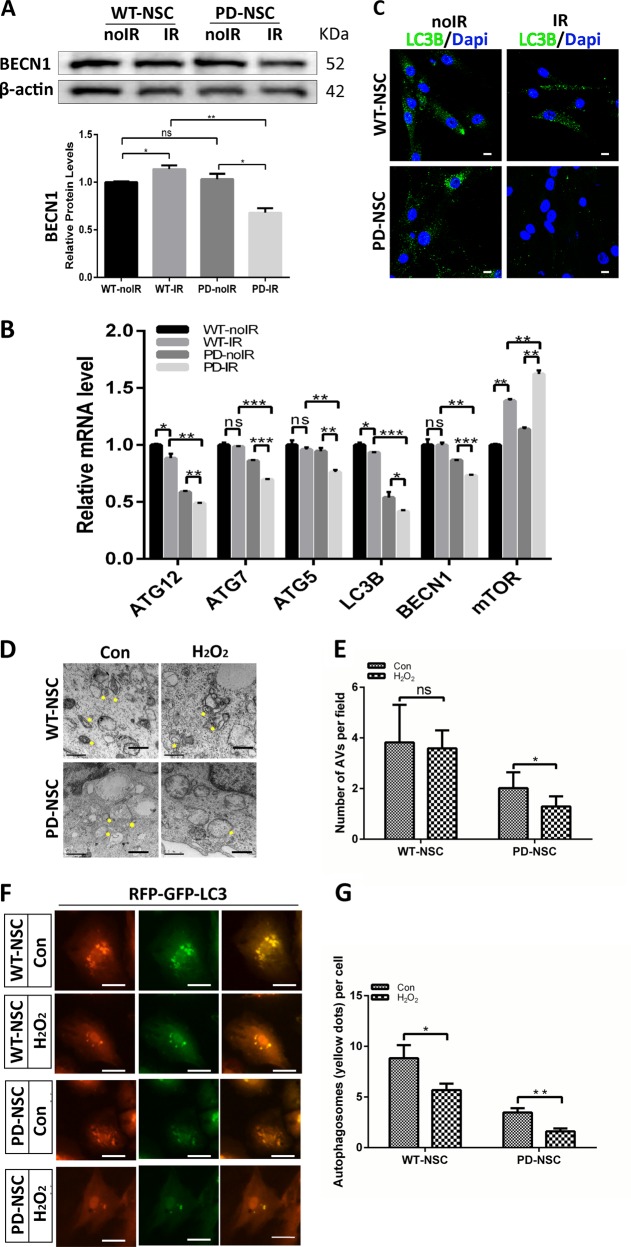
PD-NSCs showed autophagy dysfunction during the aging process after IR. **a** BECN1 expression in PD-NSCs after irradiation treatment was significantly decreased as revealed by western blot and densitometric analysis. **b** The mRNA expression of several genes linked with autophagy function, such as *BECN1*, *LC3B*, *ATG5*, *ATG7*, and *ATG12*, was consistently decreased in PD-NSCs with IR treatment. **c** The expression of LC3B in PD-NSCs with irradiation was further reduced as detected by immunostaing. Scale bar: 10 μm. **d**, **e** The double membrane structures of autophagy vacuoles in PD-NSCs after oxidative stress were decreased as analyzed by electron microscope under a JEM 1010 transmission electron microscope. NSCs were treated with H_2_O_2_ and incubated for 48 h, and then were fixed and stained. The yellow asterisk indicated autophagic vacuoles, including autophagosomes. Scale bar: 0.5 μm. **f**, **g** Distribution of GFP-LC3 in PD-NSCs changed dramatically under post-H_2_O_2_ conditions. After cells were treated with H_2_O_2_ for 48 h, they were infected with RFP-GFP-LC3 virus. Scale bar: 50 μm. All the data were expressed as mean ± SD from three independent experiments. Statistical analysis was performed by Student’s *t*-test, *P < 0.05; **P < 0.01; ***P < 0.001; ns not statistically significant. All data were obtained from at least three independent experiments

Autophagy, which could degrade misfolded proteins or injured organelles, is considered as a key survival mechanism in aging process^24–29^. Therefore, it is intriguing to explore whether autophagic function is altered in PD-NSCs and attributable to aforementioned phenotypes. We observed the downregulation of autophagy-related gene *BECN1* in PD-NSCs after IR exposure both by western blot and Q-PCR (Fig. 5a, b). Other key players in maintaining normal function of autophagy such as ATG5, 7, 12, LC3B et al. were also downregulated by IR in PD-NSCs while the expression of mTOR, the inhibitor of autophagy, was significantly augmented (Fig. 5b). Consistently, the number of cytosolic autophagosome puncta revealed by LC3B staining decreased significantly in PD-NSCs by IR treatment (Fig. 5c). We also observed the same deficient phenotypes in PD-S-NSCs (Fig. S7). Furthermore, the results from electron microscopy analysis confirmed that there was little change in the number of autophage vacuoles (AVs) in WT-NSCs after oxidative stress treatment, while marked reduction of AVs was induced in PD-NSCs (Fig. 5d, e). We then assessed autophagic function using RFP-GFP-LC3 vector. The number of autophagosomes (as indicated by yellow puncta) decreased after treatment with H_2_O_2_, and this tendency was more significant in PD-NSCs compared with WT-NSCs (Fig. 5f, g). These data strongly suggested that autophagy induction was blocked in PD-NSCs after stress exposure.

**Fig. 6:**
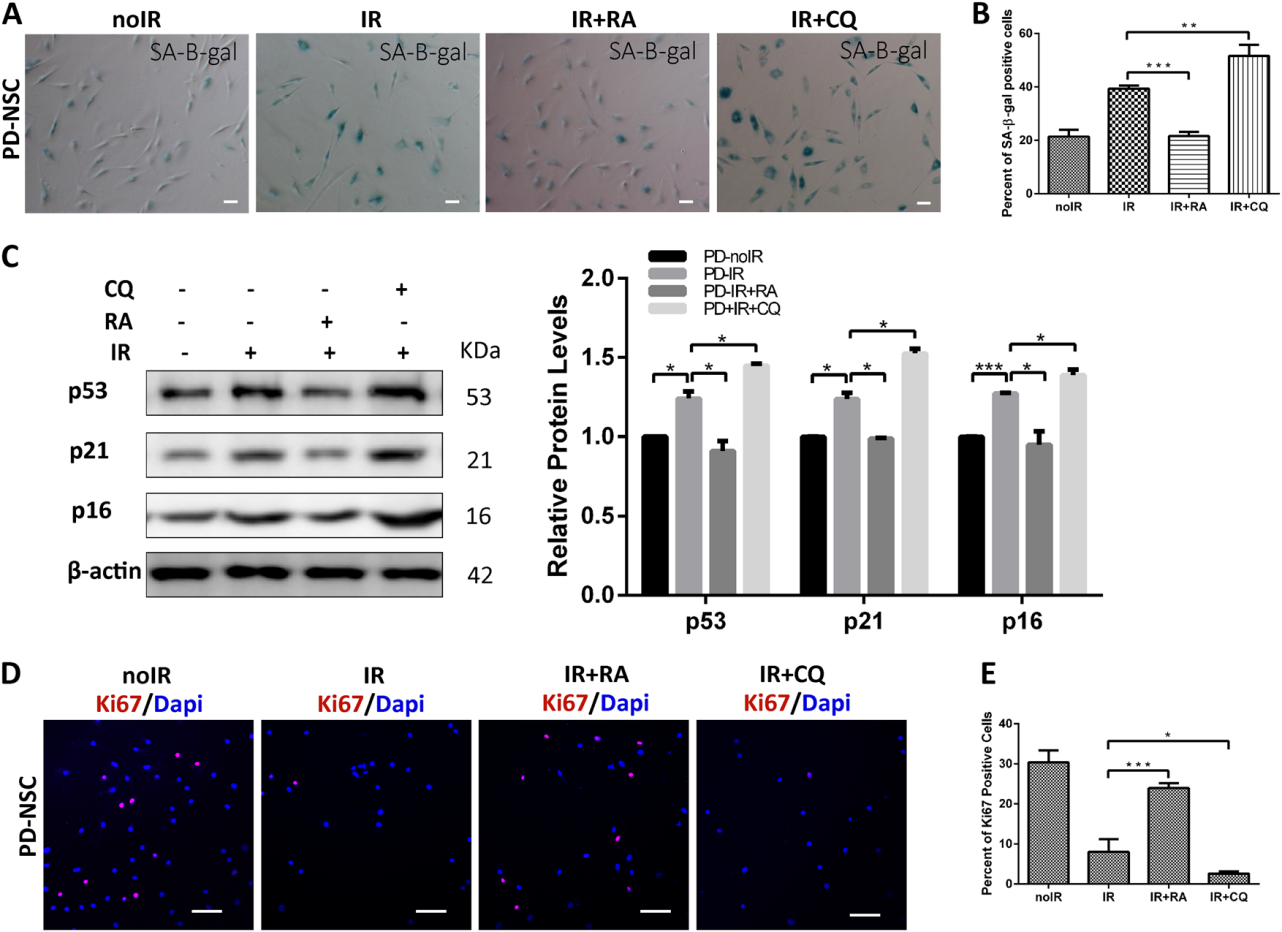
Autophagy was implicated in the IR-induced premature aging of PD-NSCs. **a**, **b** Cell senescence induced by irradiation was detected by SA-β-gal staining in the presence of rapamycin or chloroquine. Cells were exposed to 10 Gy X-ray in the presence of 0.04 μM rapamycin or 3 μM chloroquine, incubated for 48 h, and then stained with SA-β-gal. **c** p16, p21, and p53 expression from rapamycin- or chloroquine-treated NSCs were detected by western blotting and densitometry. **d**, **e** The effect of rapamycin or chloroquine on Ki67 expression was visualized by immunostaining. Data represent as the mean ± SD of three independent experiments. Statistical analysis was performed by Student’s *t*-test, *P < 0.05, **P < 0.01, ***P < 0.001. All data were obtained from at least three independent experiments. Scale bar = 100 μm

To further elucidate whether autophagic dysfunction contributes to the premature aging of PD-NSCs, we treated PD-NSCs with the activator and inhibitor of autophagy, rapamycin and chloroquine respectively while exposed to IR. Rapamycin almost reversed the senescent phenotype of PD-NSCs, whereas chloroquine further aggravated it (Fig. 6a, b). Also rapamycin could resist the premature aging of PD-S-NSCs (Fig. S5). Consistently, in PD-NSCs, the expression of aging-related genes, *p53*, *p21*, and *p16* were reduced by rapamycin and enhanced by chloroquine (Fig. 6c). Moreover, IR-induced compromised proliferation could be largely rescued by rapamycin while chloroquine almost abolished that (Fig. 6d, e). These data jointly demonstrated that autophagic dysfunction could endow the precocious aging phenotypes in PD-NSCs.

### Autophagic dysfunction in PD-NSCs induced by IR is attributable to the downregulation of SIRT1

**Fig. 7:**
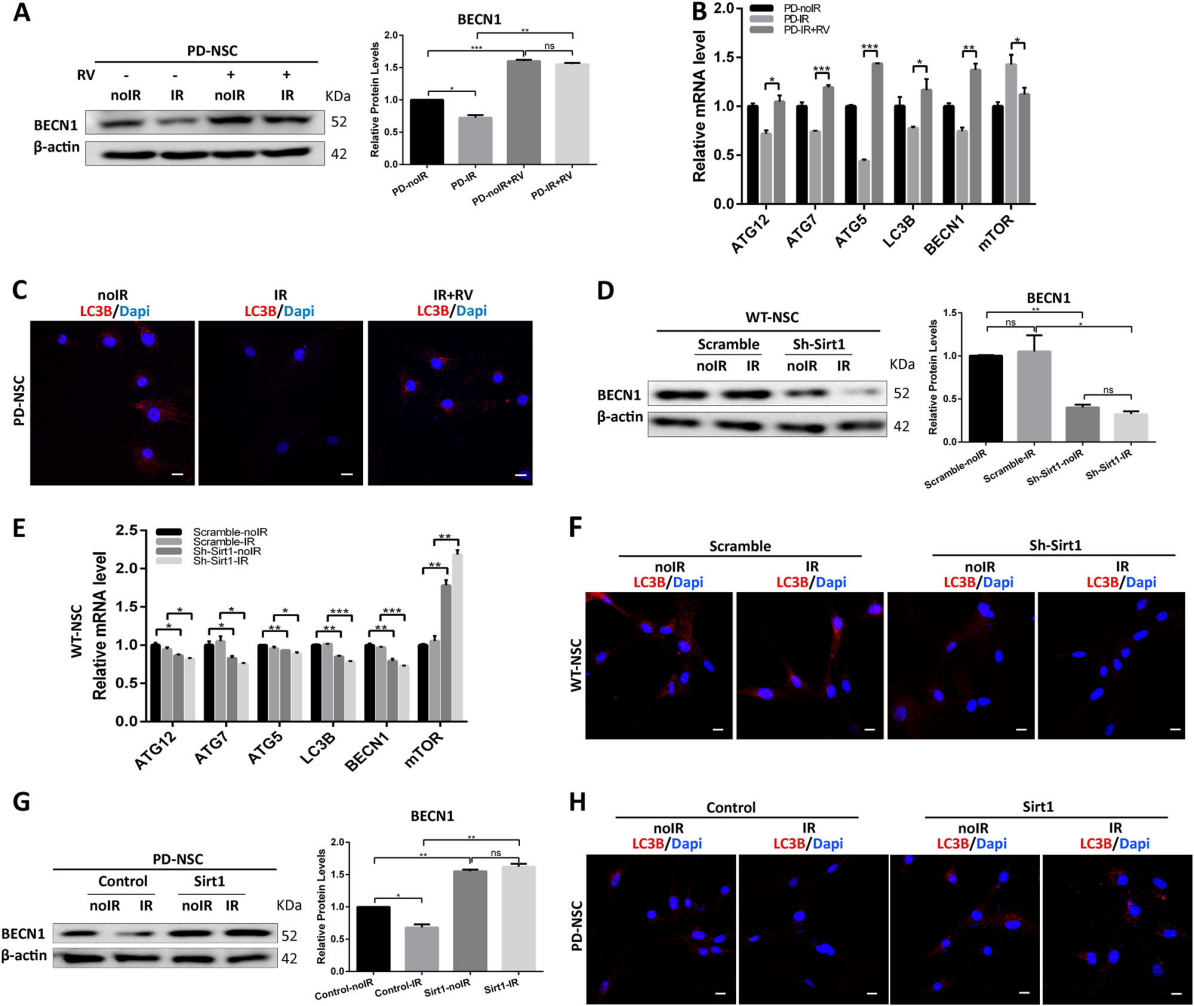
SIRT1 could regulate autophagy in the aging process of PD-NSCs. **a** Reduction of BECN1 protein levels in PD-NSCs by IR treatment was restored by 3 μM resveratrol treatment, analyzed by western blotting and densitometry. **b** The decrease of mRNA expression of autophagy-related genes, such as *BECN1*, *LC3B*, *ATG5*, *ATG7*, and *ATG12* in PD-NSCs treated with IR was prevented by administration of 3 μM resveratrol. **c** The suppression of LC3B expression in PD-NSCs after IR treatment was restored in the presence of 3 μM resveratrol. **d** The expression of BECN1 in WT-NSCs with Sirt1 knockdown decreased significantly compared with control group, analyzed by western blotting and densitometry. **e** The mRNA expression of autophagy-related genes, such as *BECN1*, *LC3B*, *ATG5*, *ATG7*, and *ATG12* in WT-NSCs treated with IR was further reduced when Sirt1 expression was inhibited. **f** The expression of LC3B decreased by knockdown of Sirt1 in WT-NSCs after IR treatment. **g** The decline of BECN1 expression in PD-NSCs after IR was inhibited by overexpression of Sirt1, analyzed by western blotting and densitometry. **h** The reduction of LC3B in PD-NSCs post IR was alleviated after Sirt1 was upregulated. The data were expressed as mean ± SD from three independent experiments. *P < 0.05, **P < 0.01, ***P < 0.001, ns not statistically significant, Student’s *t*-test. All data were obtained from at least three independent experiments. Scale bar = 100 μm

Recent studies have reported that SIRT1 is capable of stimulating basal activities of autophagy^30–33^. Therefore, we sought to investigate whether loss of SIRT1 in PD-NSCs could lead to impairment of autophagy. When treated with resveratrol, IR-induced downregulation of BECN1 was almost reversed (Fig. 7a). Consistently, Q-PCR analysis revealed that in PD-NSCs challenged by IR, a spectrum of autophagy-related genes, such as *ATG5/7/12* and *LC3B* responded to resveratrol similarly as BECN1 while mTOR expression was reduced (Fig. 7b). Consequently, LC3B expression was also augmented by resveratrol (Fig. 7c). These data indicated that activation of SIRT1 attenuated the IR-induced autophagy dysfunction.

We further found that suppression of SIRT1 attenuated BECN1 protein level without IR treatment while IR could further reduce the BECN1 expression (Fig. 7d), which basically represented the similar tendency for a pan of autophagic genes (Fig. 7e). Similarly, SIRT1 knockdown itself reduced LC3B expression while IR treatment simply aggravated it (Fig. 7f). Furthermore, we found that upregulation of SIRT1 in PD-NSCs led to increased level of BECN1 and the IR-induced reduction of BECN1 was markedly recovered (Fig. 7g). We also found that senescence-induced suppression of LC3B was attenuated in PD-NSCs with SIRT1 upregulation (Fig. 7h). All these results proved that SIRT1 downregulation endowed the autophagic dysfunction in PD-NSCs, especially under genotoxic stress.

### SIRT1 can directly interact with autophagy machinery and regulate autophagic function

**Fig. 8:**
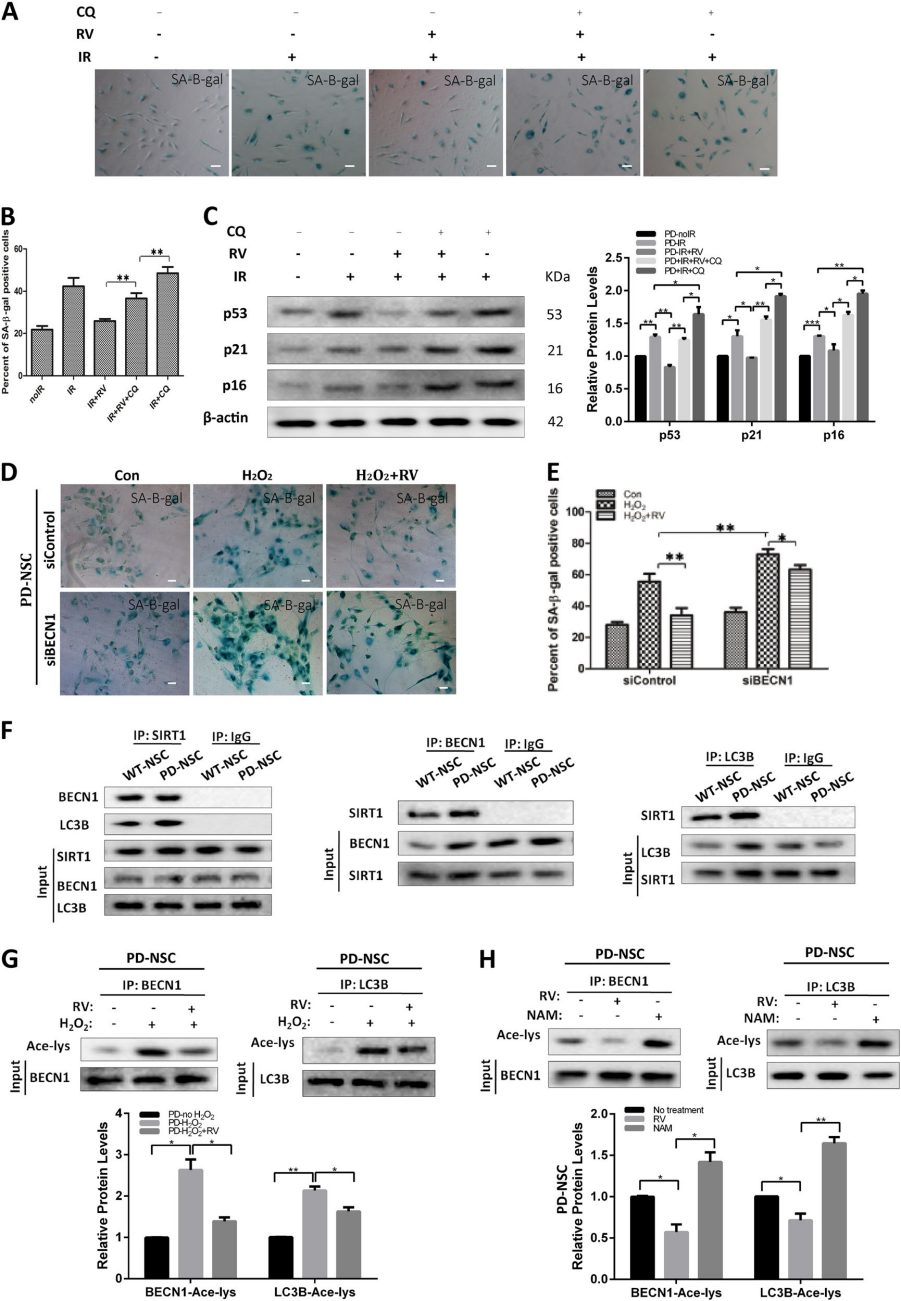
SIRT1 regulated autophagy through physically interacted with and deacetylated BECN1 and LC3B in PD-NSCs. **a**–**c** Cell senescence of PD-NSCs treated with or without resveratrol or chloroquine was measured, including SA-β-gal staining (**a**, **b**) and expression of aging-related genes (**c**), analyzed by western blotting and densitometry. Cells were exposed to 10 Gy X-ray in the presence of 3 μM resveratrol or 3 μM chloroquine, and then incubated for 48 h. **d**, **e** Images of cellular senescence in PD-NSCs after knockdown of BECN1 were visualized using SA-β-gal assay. Cells were transfected with siBECN1, and then exposed to 10 Gy X-ray in the presence of 3 μM resveratrol, incubated for 48 h. **f** The interaction between SIRT1 and autophagy-related genes, *LC3B* and *BECN1*, in PD-NSCs. Cell lysates were immunoprecipitated with anti-SIRT1 or control IgG antibody; and then, reciprocally probed with anti-BECN1 and anti-LC3B. Cell lysates were immunoprecipitated with anti-BECN1, anti-LC3B, or control IgG antibody; and then, reciprocally probed with anti-SIRT1. **g** Western blot and densitometric analysis of acetylation changes of BECN1 and LC3B after IR treatment in PD-NSCs. BECN1 and LC3B were immunoprecipitated from the total cell lysate of NSCs by BECN1 and LC3B antibody; and then, western blots were probed with anti-acetylated lysine antibody. **h** Western blot and densitometric analysis of acetylation changes of BECN1 and LC3B after PD-NSCs were treated with SIRT1 activator resveratrol (3 μM) and SIRT1 inhibitor nicotinamide (5 mM). BECN1 and LC3B were immunoprecipitated from the total cell lysate of NSCs by BECN1 and LC3B antibody; and then, western blots were probed with anti-acetylated lysine antibody. Data of the graph were expressed as the mean ± SD. *P < 0.05, **P < 0.01, ***P < 0.001, Student’s *t*-test. All data were obtained from at least three independent experiments. Scale bar = 100 μm

We treated cells with SIRT1 activator resveratrol and autophagy inhibitor chloroquine combined with IR exposure. Resveratrol could partially reverse the senescence induced by IR, whereas this effect was largely abolished by chloroquine (Fig. 8a, b). Meanwhile, chloroquine itself could further aggravate the IR-induced senescence of PD-NSCs. Consistently, the resveratrol-induced reduction of aging-related genes, *P53*, *P21*, and *P16*, in PD-NSCs upon IR treatment was inhibited by chloroquine exposure (Fig. 8c). Additionally, resveratrol could significantly attenuate H_2_O_2_-induced augment of premature aging in PD-NSCs, which was largely blocked by disrupting the autophagy function through BECN1 knockdown (Fig. 8d, e). These results suggested that IR-induced autophagic dysfunction was a downstream effect of SIRT1 downregulation.

To further confirm that SIRT1 might interact directly with some components of autophagy machinery, we assessed the interaction between SIRT1 and some essential autophagy genes, such as *BECN1* and *LC3B* by immunoprecipitation. As shown in Fig. 8f, there was remarkable interaction of SIRT1 with either BECN1 or LC3B. Some studies proved that SIRT1, as a deacetylase, could enhance basal autophagy by deacetylating ATG5, ATG7, and ATG8^33^. Inactivation of BECN1 protein induced by augmented acetylation could be achieved by SIRT1 inhibition^34^. Our results showed that BECN1 and LC3B acetylation levels significantly increased after H_2_O_2_ exposure, which could be prevented partially by resveratrol (Fig. 8g). We also found that treatment of cells with SIRT1 inhibitor nicotinamide had an enhanced effect on BECN1 and LC3B acetylation (Fig. 8h). These results suggested that stress-induced downregulation of SIRT1 in PD-NSCs could cause autophagy deficits by increasing acetylation levels of autophagy proteins, which eventually lead to premature aging of PD-NSCs.

### PD iPSCs display deficiency in the development of brain organoids

Recently, human brain organoids were used to recapitulate brain development and disease progress^35,36^. To explore whether the defects we observed at stage of NSCs affect the development of brain organoids, we investigated the differences between WT and PD organoids. As shown in Fig. S3, organoids derived from WT iPSCs showed smooth edges and optically translucent surface tissue consistent with neuroectoderm, while organoids from PD iPSCs lacked optical clearing and translucent neuroectoderm and large amounts of cell debris accumulated, suggesting failed neural induction. Moreover, the sizes of PD organoids were much smaller than WT group, which further proved that PD iPSCs displayed developmental drawbacks of brain organoids.

## Discussion

Several PD-related genes had been shown to play roles in embryonic or adult neurogenesis. The deficiency in LRRK2, VPS35, SNCA, and PINK1 led to deregulated embryonic neurogenesis and neurite outgrowth^37–40^. Adult neurogenesis was also affected in both PD patients and PD animal models with mutant forms of PD-related genes. NSCs from transgenic mice expressing mutant LRRK2 exhibited deficient proliferation and reduced newborn neurons^15,16^. NSCs carrying the SNCA mutation manifested reduced proliferation, impaired neurogenesis, and increased cell death^11–14^. Other PD-related genes, such as *VPS35*, *Parkin*, *PINK1*, and *DJ-1* et al., also played important roles in the proliferation or maintenance of NSCs, neural progenitor cells, or iPSCs. Postmortem brain tissues of PD patients were found to have decreased proliferating cell nuclear antigen (PCNA)-positive cells as well as reduced nestin-positive precursor cells in olfactory bulb and dentate gyrus^41,42^. Liu et al. found that PD patient-derived NSCs, which carried LRRK2 (G2019S) mutation, showed increased susceptibility to proteasomal stress and passage-dependent deficiencies in proliferation and neuronal differentiation^10^. Taken together, these studies strongly suggested the potential correlation between deregulated neurogenesis and pathophysiology of PD. In our studies, we found that early-onset PD patient-derived NSCs possessed susceptibility of premature aging in respond to IR exposure. These findings provide compelling evidence to support the role of early developmental deficits in the pathogenesis of PD. However, the regulatory mechanisms underlying PD-NSCs senescence as well as its correlation with PD pathology remain largely ambiguous.

We generated iPSCs lines from two patients with idiopathic early-onset PD. We identified an insertion mutation in PLA2G6 and an essential splice-site mutation in PACRG respectively in samples from the two patients. *PLA2G6* is the causative gene for PARK14-linked parkinsonism, which is a juvenile-onset parkinsonism^43^. Mutations in PLA2G6 could cause neurodegeneration including infantile neuroaxonal dystrophy and adult-onset dystonia parkinsonism^44,45^. *PACRG* is a gene that is transcriptionally co-regulated with the parkin gene by a bi-directional promoter^46^. Many reports demonstrated that mutations in PACRG were associated with familial and also sporadic, early-onset PD^47–49^. However, we didn’t know whether the two mutations were associated with the senescence and regulatory mechanism of NSCs derived from PD patients.

In our study, we found that IR treatment could lead to downregulation of SIRT1 in PD-NSCs. This phenomenon is similar to that observed in endothelial cells whereby cellular senescence is attributable for the development of age-related diseases, such as atherosclerosis^20^. Our results collectively suggested that SIRT1 played an essential role in controlling cellular senescence of NSCs. In this study, we observed that PD-NSCs were more susceptible to autophagy dysfunction in response to IR. The senescence could be largely reversed by boosting the autophagic function and otherwise aggravated by further blocking the autophagy. We further demonstrated that stress-induced SIRT1 reduction could account for the autophagic dysfunction and eventually lead to cellular senescence of PD-NSCs. It is of paramount importance to determine whether these mutations carried by patients have anything to do with the susceptibility of SIRT1 and autophagy to stresses at the stage of NSCs, which, in turn, contribute to pathogenesis of PD.

In summary, we demonstrated that the NSCs, which were derived from early-onset PD patients, manifested susceptibility to various stresses and were prone to become more senescent. Also, we outlined a relatively clear picture of the molecular mechanism of PD pathology from different perspective that dysregulation of SIRT1 was a pivotal player in endowing NSCs the susceptibility to stress by modulating autophagy activity. Based on this study, in combination with other previous reports, it is tempting to hypothesize that developmental defects, which might contribute to constraining the pool size of DA neuron number when growing into adulthood, could have profound impact on the onset timing and the severity of progression of PD. To consolidate this hypothesis, there are still a few critical questions to be addressed. First of all, it is essential to know whether the deficits we observed at the stage of NSCs will only preferentially affect the genesis of DA neurons. Second, it needs to be clarified how the mutations carried by PD patients regulate the SIRT1 and autophagy as well as the subsequent signaling pathways.

Owing to the limited number of early-onset PD patients, we were not 100% sure that the premature aging of PD-NSCs and regulatory mechanisms could be repetitive in all idiopathic early-onset PD patients. However, these results indicated the possibility that the senescent phenotypes, deficient SIRT1 expression, and dysfunctional autophagy in PD-NSCs might contribute to the defects of dopaminergic neuron pools and other neurodegenerative disorder associated with PD pathology. We can use this knowledge to formulate a novel paradigm of analysis to better predict the onset and the progression of PD and provide a preventive intervention toward the cure of the disease.

## Materials and methods

### Clinical data for patient-specific iPSC lines

In this study, we recruited individuals from Han Chinese population. Individuals in each group were also selected so that no significant bias in gender or age occurred.

We obtained PD patients’ skin tissues from Ruijin Hospital affiliated to Shanghai Jiaotong University School of Medicine. The iPSC lines were generated from two subjects with idiopathic early-onset PD under informed consent. Subject 1 was a 35-year-old male and carried a rare homozygous insertion within PLA2G6 c.28dupA (p.T10fs), with no family history of PD. The age of onset of this PD patient with PLA2G6 mutation was 28 years. He had a score of 2.5 on the Hoehn and Yahr scale and showed serious resting tremor. Subject 2 was a 21-year-old man and diagnosed with idiopathic PD at age 19, with no family history of the disease. He had a potentially deleterious PACRG splice-site mutation, c.292-1G > A mutation. He had a score of 3.5 on the Hoehn and Yahr scale, indicating severe disability.

The healthy individual in our study was a 28-year-old male and had no history of neurological disease.

### Generation of human skin fibroblast-derived iPSCs

Fibroblasts were isolated and amplified, then were infected with retroviruses expressing OCT4, SOX2, Nanog, and KLF4, and maintained in hESC medium on iMEF feeder cells. After 30 days induction, the skin cells were reprogrammed into iPSCs. Ten to twenty independent iPSC lines per individual were generated, totaling 50 iPSC lines. Two iPSC lines per patient were thoroughly characterized and sustained long-term passaging (>20 passages) and then differentiated into neural progenitors for detailed analysis.

### iPSC culture and neural induction

iPSCs were plated onto a feeder layer of irradiated mouse embryonic fibroblasts in embryonic stem (ES) cell medium containing 80% Dulbecco’s modified Eagle’s medium (DMEM)/F12, 20% knockout serum replacement, 0.1 mM β-mercaptoethanol, 1 mM glutamine, and 1% nonessential amino acids, supplemented with 10 ng/ml bFGF. The iPSCs were fed every day until ready to passage. To passage iPSCs, cells were incubated with 1 mg/ml collagenase IV in DMEM/F12 for about 15–30 min at 37 °C. Then, the colonies were collected, gently dissociated into small clusters, and plated in fresh medium.

The induction of neural precursor cells was based on a previous report^50^. Briefly, after digestion and trituration, cells were resuspended in ES cell medium without FGF-2 for 6 days as floating embryonic bodies (EBs). Then, medium was switched to N2 medium consisting of DMEM/F12, 1% N2 supplement, 1% nonessential amino acids, 2 µg/ml heparin, and 10 ng/ml bFGF for 10 days. To separate the rosette clusters from the differentiating EBs, the cells were incubated with 0.1 mg/ml dispase at 37 °C for 15–20 min. Then, the rosette clumps were isolated, gently triturated, and plated in fresh N2 medium as neural stem cells. Culture was fed every 2 or 3 days. Cells were subcultured once a week by triturating the neurospheres after digestion with dispase. For adhesion culture, the NSCs were cultured in matrigel-coated plates.

To analyze the differentiation potential, NSCs were cultured on poly-ornithine/laminin-coated plates in N2 medium without bFGF for 10 days. Medium were changed every 3 days.

### Irradiation treatment

Cells were seeded at 1 × 10^4^ cells/ml in 24-well plates (Corning Incorporated, USA), treated with 10 Gy X-ray irradiation, and incubated at 37 °C until the collection time points.

### Chemical molecule treatment

Cells were seeded at 1 × 10^4^ cells/ml in 24-well plates, pretreated with chemical molecules, and then exposed to 10 Gy X-ray irradiation followed by incubation at 37 °C until the collection time points. The concentration of resveratrol (Sigma) was 3 μM. The concentration of rapamycin (Sigma) was 0.04 μM. The concentration of chloroquine (Sigma) was 3 μM. The concentration of SB203580 (Sigma) was 20 μM. The concentration of nicotinamide (Sigma) was 5 mM.

### ROS measurement

To measure intracellular ROS, cells were incubated in 2′,7′-dichlorofluorescein diacetate (DCF-DA) solution (Genmed Scientifics) at 37 °C for 20 min and then moved to the Nikon E800 fluorescence microscope (Nikon, Japan). Experiments were performed in triplicates from three independent trials.

### SA-β-gal assay

Cellular senescence was determined using the SA-β-gal staining Kit (Genmed Scientifics). Briefly, after 12 h incubation at 37 °C, the blue stained cells from 10 different fields were captured with an optic microscope and were counted with results presented as a percentage of positive cells.

### CCK8 assay

For the cell viability assessment, NSCs derived from iPS cells were plated onto 96-well plates (about 3 × 10^3^ cells/well). Twenty-four hours after seeding the cells, each well was treated with 10 Gy X-ray irradiation. At the end of each time point, 10 µl CCK8 solution (Dojindo Laboratories) was added to each well, and the plates were incubated for an additional 2.5 h at 37 °C. The absorbance of each plate at 450 nm represented a direct correlation with the cell number in this analysis and was measured using a standard microplate reader (Thermo Scientific Varioskan Flash, USA).

### Immunofluorescence analysis

Cells were fixed in 4% paraformaldehyde, then rinsed with phosphate-buffered saline (PBS) three times. Cells were blocked in 3% normal donkey serum in PBS for 1 h. Cells then were incubated with primary antibody diluted in 3% normal donkey serum in PBS at 4 °C overnight. After that, cells were rinsed with PBS containing 0.1% Triton X-100 (Sigma) three times and incubated with secondary antibody for 1 h. Cell nucleus were stained with 4′,6-diamidino-2-phenylindole (Invitrogen) for 10 min and slides were washed three times in PBS containing 0.1% Triton X-100 (Sigma). Images were obtained on a Leica TCS SP2 confocal fluorescence microscope.

### Protein extraction and western blotting

Cells were harvested and lysed in lysis buffer for protein extraction. Protein concentrations were measured by BCA protein assay kit (Thermo Scientific). Proteins were separated by SDS-polyacrylamide gel electrophoresis and transferred onto polyvinylidene fluoride membranes. The membranes were incubated with primary antibodies: rabbit anti-SIRT1 (Millipore), anti-BECN1 (Abclonal Technology), anti-LC3B (Cell Signaling Technology), anti-p16 (Abclonal Technology), anti-p21 (Abclonal Technology), anti-p53 (Abclonal Technology), and mouse anti-β-actin (Sigma) followed by incubation with horseradish peroxidase (HRP)-conjugated secondary antibodies (HRP-donkey-anti-rabbit and HRP-donkey-anti-mouse) (Santa Cruz Biotechnology). Signal was detected by an ECL kit. The experiments were performed three times.

### Immunoprecipitation and acetylation detection

Cells were lysed in lysis buffer followed by the determination of protein concentration. In order to analyze the direct interaction between SIRT1 and autophagy-related genes LC3B or BECN1, SIRT1 or LC3B, or BECN1 was pulled down from total cell lysate with conjugated anti-SIRT1 or LC3B or BECN1-agarose beads (Santa Cruz Biotechnology), followed by LC3B or BECN1 or SIRT1 detection with rabbit anti-LC3B or BECN1 or SIRT1 antibody (Cell Signaling Technology). To analyze LC3B and BECN1 acetylation, LC3B or BECN1 was pulled down from total cell lysate with conjugated anti-LC3B or BECN1-agarose beads (Santa Cruz Biotechnology), followed by acetylation detection with rabbit anti-acetyl lysine antibody (Cell Signaling Technology). The experiments were performed three times.

### Real-time quantitative PCR

For Q-PCR, total RNA was reverse-transcribed using PrimeScriptII 1st Strand cDNA Synthesis Kit (TaKaRa). Detection of target gene expression was performed by using a SYBR Premix Ex Taq kit (TaKaRa) and Fluorescence quantitative PCR instrument (Applied Biosystems 7500 Fast Real-Time PCR Systems, Life Technologies, CA, USA). Primers were designed using Primer 3 online and experimentally validated. The relative expression levels were determined by employing the 2^−ΔΔCT^ method normalized to housekeeping gene *GAPDH*. Experiments were repeated in triplicate.

### RNA interference targeting SIRT1

The anti-SIRT1 shRNA was used for SIRT1 downregulation (Sh-Sirt1). The plasmid carrying wild-type SIRT1 cDNA was served for SIRT1 upregulation. Recombinant vectors together with three lentiviral packaging plasmids (at a 10:5:3:2 ratio) were transfected into 293T cells using Lipofectamine 2000 (Invitrogen). Serial dilution methods were performed to determine the lentivirus titer. The lentivirus were used to infect NSCs at appropriate titers. These cells were harvested 96 h after infection, and real-time PCR and western blot analysis were performed to determine the efficiency of SIRT1 downregulation and upregulation. The experiments were repeated three times.

### Transfection of cells with Beclin 1 small interference RNA

Cells were transiently transfected with small interference RNA (siRNA) of Beclin 1 (sense, GGAGCCAUUUAUUGAAACUTT; antisense, AGUUUCAAUAAAUGGCUCCTT) or control nonspecific siRNA (sense, UUCUCCGAACGUGUCACGUTT; antisense, ACGUGACACGUUCGGAGAATT) using FuGENE HD Transfection Reagent (Promega). Forty-eight hours after transfection, cells were exposed to various treatments as specifically indicated.

### Autophagy detection

To further determine the induction of autophagy, the development of acidic vesicular organelles was tested using adenoviral vectors with mRFP-GFP-LC3, which were obtained from HanBio Technology Co. Ltd. (HanBio). After treatment, autophagosomes and autolysosomes were observed under confocal microscopy (Leica TCS SP8, Germany). The total number of puncta per cell was counted. To confirm the autophagosome structures of autophagy, cells were fixed with a solution containing 3% glutaraldehyde plus 2% paraformaldehyde in 0.1 M PBS (pH = 7.3) for 1 h for further analysis by transmission electron microscopy.

### Statistical analysis

Data were analyzed by Student’s *t*-test and one-way analysis of variance. Significance was accepted at *P* < 0.05. All the detailed statistical analysis was described in Supplementary Table 2.

## Supplementary information

Supplementary Information

Figure legends for Supplementary Tables

Supplementary Table 1

Supplementary Table 2
