# Supplementary material for: Stress-induced precocious aging in PD-patient iPSC-derived NSCs may underlie the pathophysiology of Parkinson’s disease
Source: Cell Death Dis. 2019 Feb 4;10(2):105. doi: 10.1038/s41419-019-1313-y (PMC6362163; doi:10.1038/s41419-019-1313-y)
Supplement: Supplementary file 1 — Supplementary Information [file 41419_2019_1313_MOESM1_ESM.docx]

**SUPPLEMENTAL FIGURES**

**
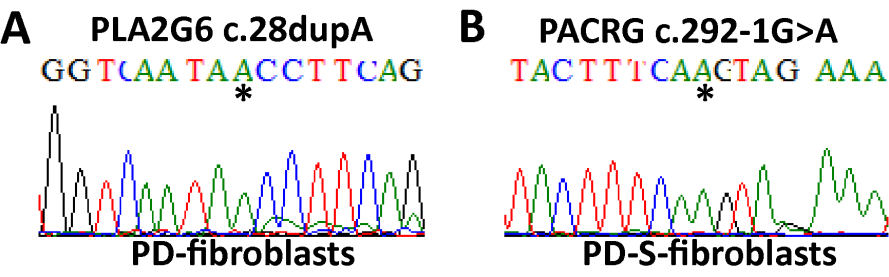
**

**Fig. S1 DNA sequencing analysis of genomic DNA from PD-fibroblasts and PD-S-fibroblasts.** **a** The chromatogram showing insertion within PLA2G6, c.28dupA (p.T10fs), in PD-fibroblasts. To confirm the mutation observed by WES, PCR products of genomic DNA from fibroblasts were sequenced. **b** The chromatogram indicated a PACRG c.292-1G>A mutation in PD-S-fibroblasts.


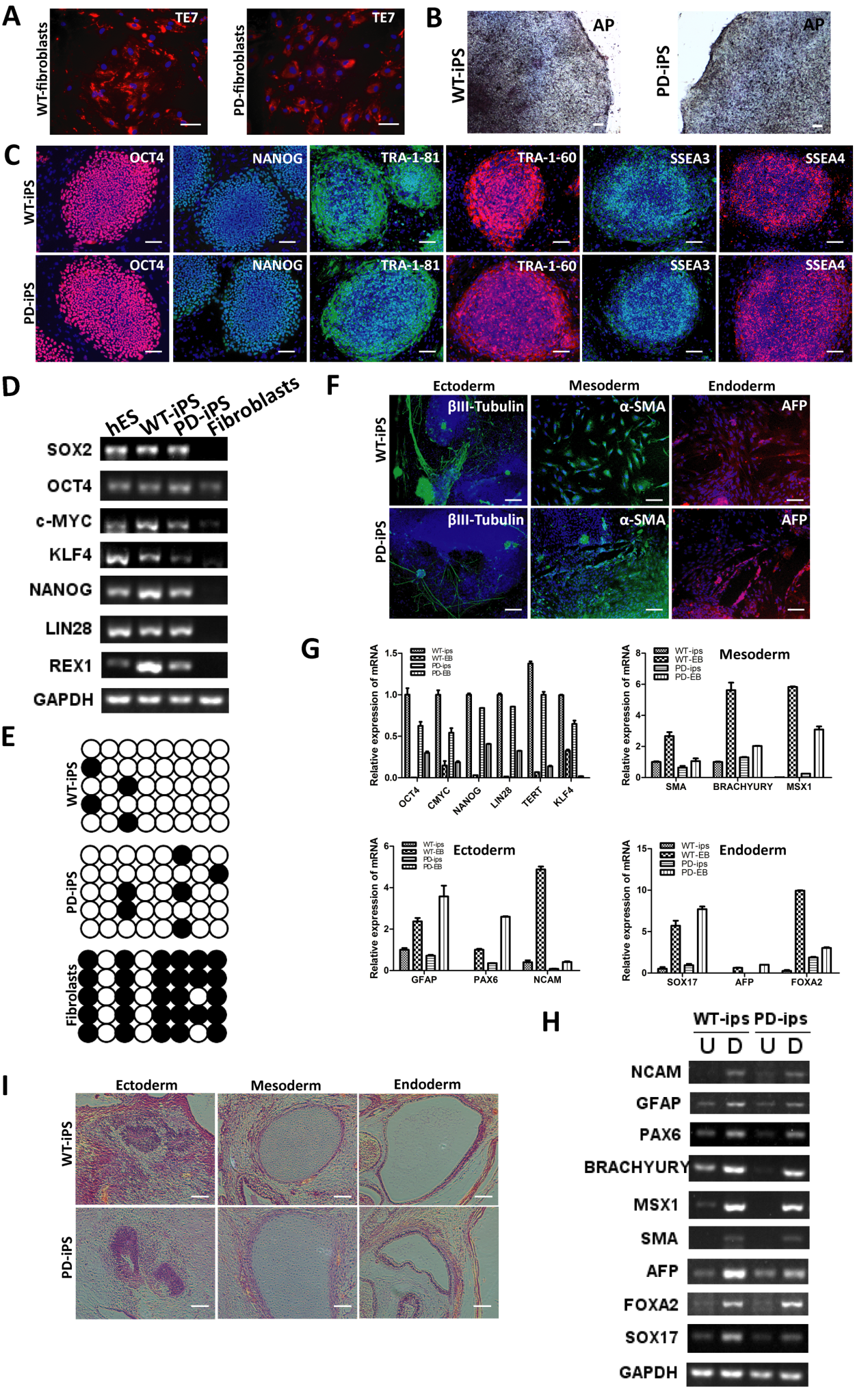


**Fig. S2 Generation and characterization of human iPSCs from early onset PD patients. a** Expression of classic human skin fibroblasts marker TE7 in fibroblast derived from early-onset PD patients and healthy human beings. Cell nuclei were counterstained with DAPI (blue). **b** PD-iPSCs and WT-iPSCs expressed pluripotency marker: alkaline phosphatase (AP). **c** PD-iPSCs and WT-iPSCs expressed typical embryonic stem cell markers SSEA-3, SSEA-4, TRA-1-60, TRA-1-81, OCT4, Nanog. Nuclei were stained with DAPI (blue). **d** RT-PCR analysis of a cohort of ES cell-marker genes for PD-iPSCs and WT-iPSCs. hESCs and fibroblasts were presented as positive and negative controls, respectively. **e** DNA methylation state of OCT4 promoter region was analyzed for PD-iPSCs, WT-iPSCs and fibrolasts. Open and closed circles represent unmethylated and methylated CpG islands, respectively. **f** In vitro differentiation of iPSCs to cells representative of each germ layer: a-fetoprotein (AFP, endoderm), b-III-tubulin (ectoderm), a-smooth muscle actin and desmin (mesoderm). **g** RT-PCR analyses of iPSCs markers and various maker genes of three germ layers in iPSCs and differentiated EBs. **h** Gene expression of ES cell-marker genes and various differentiation markers for the three germ layers in iPSCs and EBs. D: differentiation; U: undifferentiation. **i** Hematoxylin and eosin staining of teratoma derived from iPS cells. Cells were transplanted subcutaneously into a SCID mouse. Tumor developed from injection site. Scale bar is equal to 100 μm.


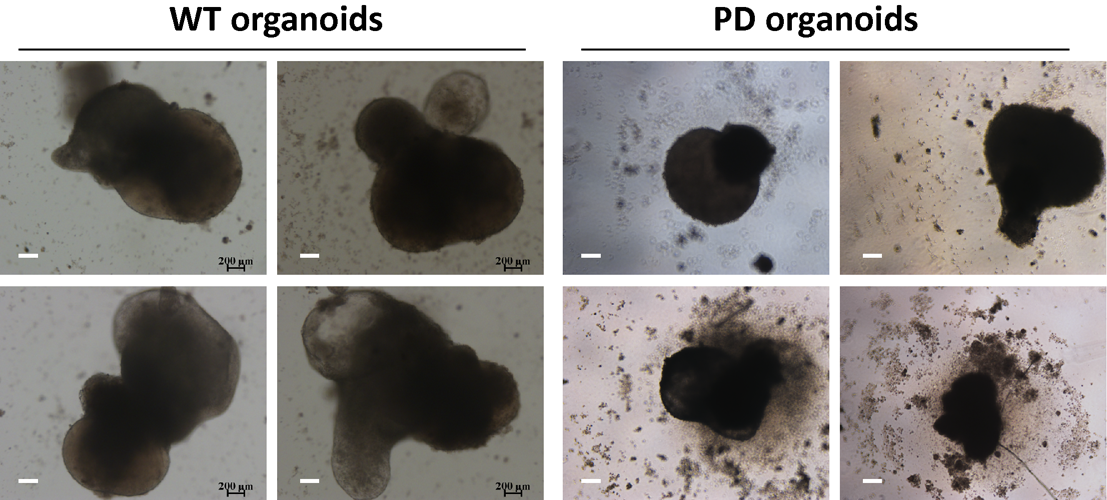


**Fig. S3 The formation of organoids was blocked in PD iPSCs group.** Representative images of human organoids at day 14 were showed. Scale bar = 200 μm.


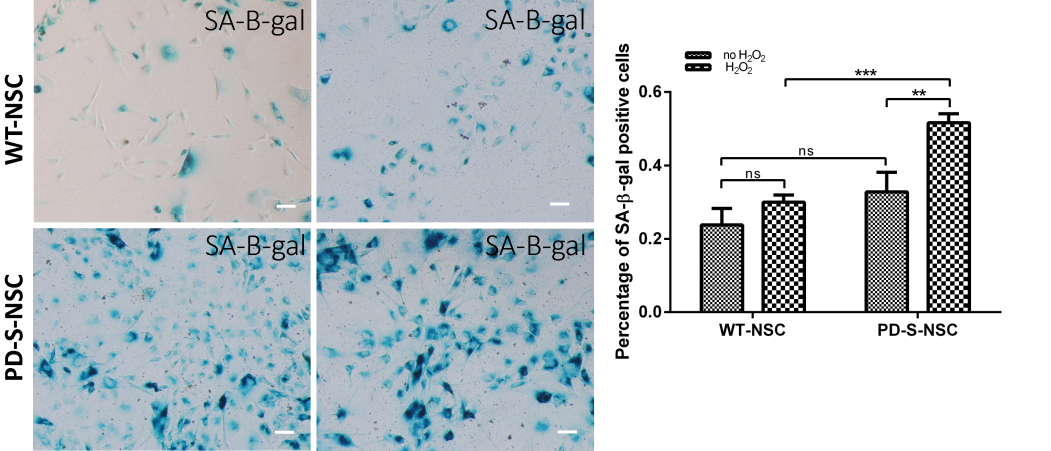


**Fig. S4 NSCs derived from the 21-year-old early-onset PD patient manifested premature aging phenotypes.** Cellular senescence was significantly aggravated by oxidative stress as evaluated with the SA-β-gal assay. Representative images from at least three independent experiments were presented. The statistical results showed the percent of SA-β-gal positive cells (%). Scale bar: 100 μm. All the data were expressed as mean ± S.D.. *P< 0.05, **P< 0.01, ***P < 0.001, ns: not statistically significant, Student’s t-test. All data were obtained from at least three independent experiments.


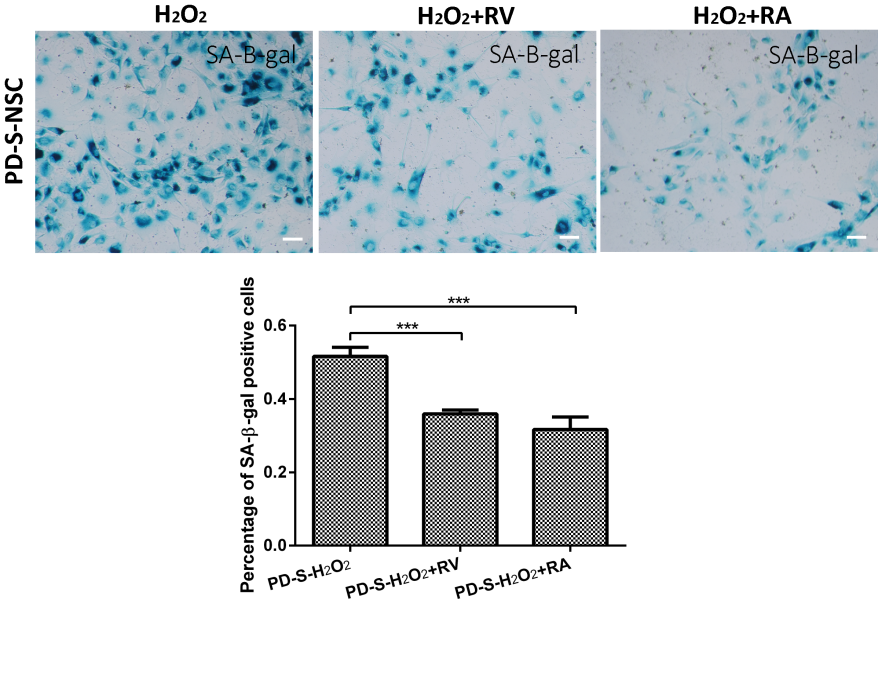


**Fig. S5 Cellular senescence in PD-S-NSCs was partially rescued by treatment with Resveratrol and Rapamycin.** NSCs derived from 21-year-old early-onset PD patient were exposed to 150 μM H2O2 in the presence of 3μM Resveratrol and 0.04μM Rapamycin. Cell senescence were detected by SA-β-gal staining. The statistical results showed the percent of SA-β-gal positive cells (%). Scale bar: 100 μm. All data were presented as results of a typical experiment from at least three independent experiments; Mean ± SD, *P< 0.05, **P< 0.01, ***P < 0.001, ns: not statistically significant, Student’s t-test. Scale bar: 100 μm.


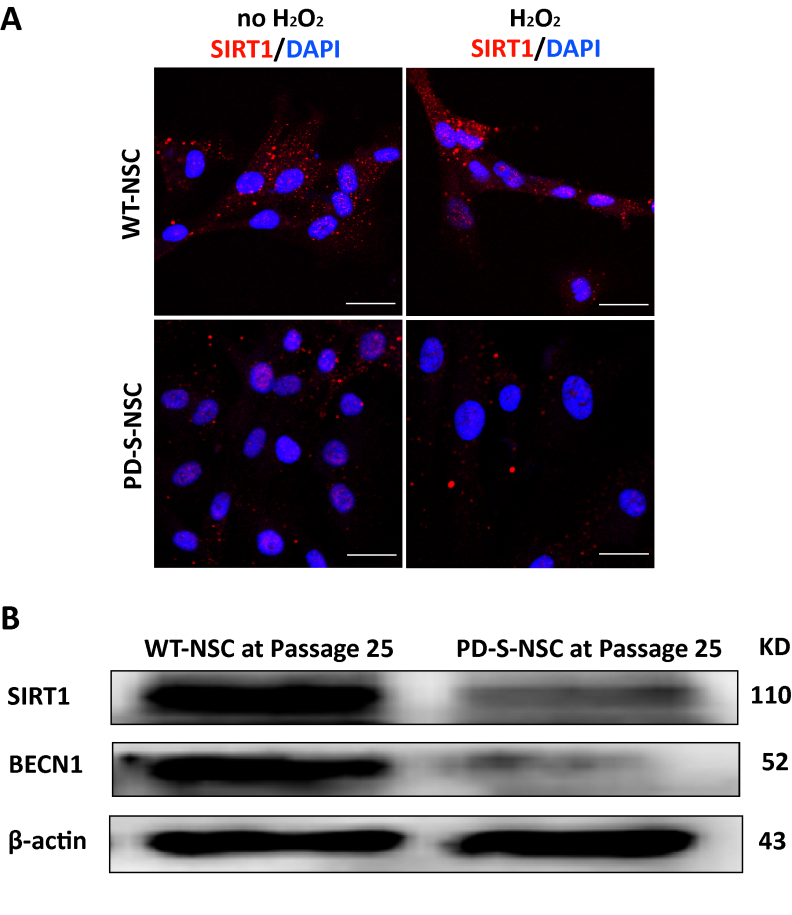


**Fig. S6 SIRT1 expression decreased in PD-S-NSCs induced by oxidative stress and extensive passaging. a** SIRT1 expression in PD-S-NSCs was significantly reduced by 100 μM H_2_O_2_ treatment for 24 hours as revealed by immunostaining. Scale bar: 50 μm. **b** SIRT1 expression was largely diminished in PD-S-NSCs after serial passaging (passage 25) as revealed by Western blotting.


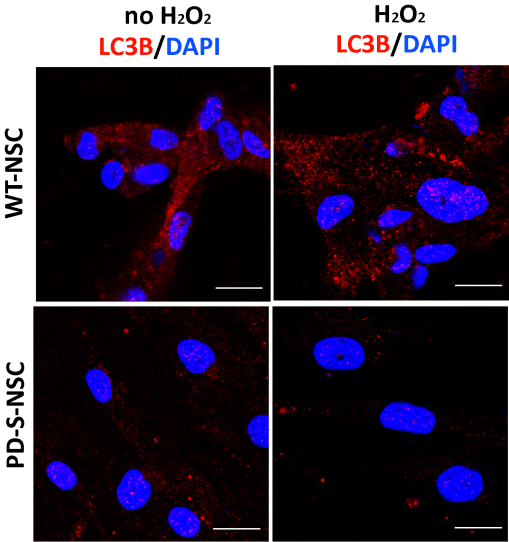


**Fig. S7 PD-S-NSCs showed reduced LC3B expression after oxidative stress treatment.** The expression of LC3B in PD-S-NSCs with 100 μM H_2_O_2_ treatment for 24 hours was further blocked as detected by immunostaining. Scale bar: 50 μm.

**SUPPLEMENTAL EXPERIMENTAL PROCEDURES**

**Generation of human brain organoids**

We applied a modified human pluripotent stem cell-derived organoid culture system based on a previous report to generate brain organoids^[1](#_ENREF_1)^. Firstly, iPSCs were dissociated to single cells to form uniformly sized embryoid bodies (EBs) in low-attachment 96-well plates (about 10000 cells per well) and feeded with hESC medium (80% DMEM-F12, 20% KSR, 1% GlutaMAX, 1% NEAA, 4 ng/ml bFGF, 50 μM ROCK inhibitor). After 5 or 6 days, EBs began to brighter and had smooth edges. To induce neuroectodermal differentiation, these EBs were cultured in neural induction medium ( DMEM-F12, 1% N2 supplement, 1% GlutaMAX, 1% NEAA, 1 μg/ml heparin, 10 ng/ml bFGF, 10 ng/ml EGF). The organoids were feeded every other day by aspirating half of the medium and adding an additional 100 µl of fresh medium. After 10 days, images of EBs were captured using an inverted microscope equipped with a camera.

**Sequences for primers used are as follows:**

| Gene | | Sequence (5’-3’) |
| --- | --- | --- |
| SOX2 | Forward primer | \| GGGAAATGGGAGGGGTGCAAAAGAGG \| \| --- \| |
|  | Reverse primer | TTGCGTGAGTGTGGATGGGATTGGTG |
| OCT4 | Forward primer | \| GACAGGGGGAGGGGAGGAGCTAGG \| \| --- \| |
|  | Reverse primer | CTTCCCTCCAACCAGTTGCCCCAAAC |
| C-MYC | Forward primer | \| GCGTCCTGGGAAGGGAGATCCGGAGC \| \| --- \| |
|  | Reverse primer | TTGAGGGGCATCGTCGCGGGAGGCTG |
| KLF4 | Forward primer | \| AGAAGGATCTCGGCCAATTT \| \| --- \| |
|  | Reverse primer | GTGGAGAAAGATGGGAGCAG |
| NANOG | Forward primer | GATTTGTGGGCCTGAAGAAA |
|  | Reverse primer | AAGTGGGTTGTTTGCCTTTG |
| LIN28 | Forward primer | \| GGATGTCTTTGTGCACCAGA \| \| --- \| |
|  | Reverse primer | CTCCTTTTGATCTGCGCTTC |
| TERT | Forward primer | \| CCTGCTCAAGCTGACTCGACACCGTG \| \| --- \| |
|  | Reverse primer | GGAAAAGCTGGCCCTGGGGTGGAGC |
| GFAP | Forward primer | TGGCCACTGTGAGGCAGAAG |
|  | Reverse primer | ACCTCCTCCTCGTGGATCTT |
| PAX6 | Forward primer | GCCAGCAACACACCTAGTCA |
|  | Reverse primer | GGGGAAATGAGTCCTGTTGA |
| NCAM | Forward primer | AACGTGAAGATCTTTCAG |
|  | Reverse primer | GATGACATCTCGGCCTTTGT |
| SMA | Forward primer | TCCTCCCGGCCACCGTACTG |
|  | Reverse primer | CGCCGCTGCTCATCGCCATA |
| BRACHYURY | Forward primer | ACCCAGTTCATAGCGGTGAC |
|  | Reverse primer | CCATTGGGAGTACCCAGGTT |
| MSX1 | Forward primer | ATGGTTCCAGAACCGCCGCG |
|  | Reverse primer | AGGCACCGTAGAGCGAGGCA |
| SOX17 | Forward primer | GCCAAGGGCGAGTCCCGTATC |
|  | Reverse primer | CGACTTGCCCAGCATCTTGCT |
| AFP | Forward primer | AGCTTGGTGGTGGATGAAAC |
|  | Reverse primer | TCTGCAATGACAGCCTCAAG |
| FOXA2 | Forward primer | GCGACCCCAAGACCTACAG |
|  | Reverse primer | GGTTCTGCCGGTAGAAGGG |
| REX1 | Forward primer | \| CAGATCCTAAACAGCTCGCAGAAT \| \| --- \| |
|  | Reverse primer | GCGTACGCAAATTAAAGTCCAGA |
| GAPDH | Forward primer | GTGGACCTGACCTGCCGTCT |
|  | Reverse primer | GGAGGAGTGGGTGTCGCTGT |
| ATG12 | Forward primer | \| CTGGAGGGGAAGGACTTACG \| \| --- \| |
|  | Reverse primer | AGTCCTTGGATGGTTCGTGT |
| ATG7 | Forward primer | \| TTCTGCAATGATGTGGTGGC \| \| --- \| |
|  | Reverse primer | AGGCTCATTCATCCGATCGT |
| ATG5 | Forward primer | \| AAGACCTTCTGCACTGTCCA \| \| --- \| |
|  | Reverse primer | GAGTTTCCGATTGATGGCCC |
| BECN1 | Forward primer | \| GACACTCAGCTCAACGTCAC \| \| --- \| |
|  | Reverse primer | CTGCCACTATCTTGCGGTTC |
| LC3B | Forward primer | \| AAGGCGCTTACAGCTCAATG \| \| --- \| |
|  | Reverse primer | CTGGGAGGCATAGACCATGT |
| mTOR | Forward primer | \| TCCGAGAGATGAGTCAAGAGG \| \| --- \| |
|  | Reverse primer | CACCTTCCACTCCTATGAGGC |

**SUPPLEMENTAL REFERENCES**

1 Lancaster, M. A. & Knoblich, J. A. Generation of cerebral organoids from human pluripotent stem cells. *Nature protocols* **9**, 2329-2340, doi:10.1038/nprot.2014.158 (2014).
