# Supplementary material for: Stress-induced precocious aging in PD-patient iPSC-derived NSCs may underlie the pathophysiology of Parkinson’s disease
Source: Cell Death Dis. 2019 Feb 4;10(2):105. doi: 10.1038/s41419-019-1313-y (PMC6362163; doi:10.1038/s41419-019-1313-y)
Supplement: Supplementary file 2 — Figure legends for Supplementary Tables [file 41419_2019_1313_MOESM2_ESM.docx]

Figure Legends for Supplementary Tables:

Supplementary Table 1: Mutations of genomic DNA of the two early-onset PD patients as revealed by whole-exome sequencing.

Supplementary Table 2: Numerical values for all experimental results. All data were obtained from at least three independent experiments; Mean ± SD, *P< 0.05, **P< 0.01, ***P < 0.001, ns: not statistically significant, Student’s t-test.
