# Supplementary material for: Stress-induced precocious aging in PD-patient iPSC-derived NSCs may underlie the pathophysiology of Parkinson’s disease
Source: Cell Death Dis. 2019 Feb 4;10(2):105. doi: 10.1038/s41419-019-1313-y (PMC6362163; doi:10.1038/s41419-019-1313-y)
Supplement: Supplementary file 4 — Supplementary Table 2 [file 41419_2019_1313_MOESM4_ESM.docx]

**Supplementary Table 2**

**Numerical values for all experimental results.** All data were obtained from at least three independent experiments; Mean ± SD, *P< 0.05, **P< 0.01, ***P < 0.001, ns: not statistically significant, Student’s t-test.

| Figure | Genotype | parameter | value | SE | P value | Cell # |
| --- | --- | --- | --- | --- | --- | --- |
| Fig. 1C | WT-NSC | Percent of Tuj1 positive | 0.42 | 0.04 | P<0.001 compared  with PD-NSC | 570 |
|  | PD-NSC | Percent of Tuj1 positive | 0.13 | 0.02 | P<0.001 compared  with WT-NSC | 548 |
|  | WT-NSC | Percent of GFAP positive | 0.50 | 0.04 | P<0.001 compared  with PD-NSC | 561 |
|  | PD-NSC | Percent of GFAP positive | 0.16 | 0.02 | P<0.001 compared  with WT-NSC | 429 |
| Fig. 1F | WT-NSC | Percent of Ki67 positive | 0.37 | 0.01 | P<0.01 compared  with PD-NSC | 137 |
|  | PD-NSC | Percent of Ki67 positive | 0.31 | 0.03 | P<0.01 compared  with WT-NSC | 179 |
| Fig. 1H | WT-NSC, noIR | Percent of SA-β-gal positive | 0.21 | 0.02 | P<0.01 compared with WT-NSC, IR; P<0.05 compared with  PD-NSC, noIR | 140 |
|  | WT-NSC, IR | Percent of SA-β-gal positive | 0.33 | 0.02 | P<0.01 compared with  WT-NSC, noIR; P<0.01 compared with PD-NSC, IR | 130 |
|  | PD-NSC, noIR | Percent of SA-β-gal positive | 0.28 | 0.02 | P<0.05 compared with  WT-NSC, noIR; P<0.001 compared with PD-NSC, IR | 133 |
|  | PD-NSC, IR | Percent of SA-β-gal positive | 0.52 | 0.04 | P<0.001 compared with PD-NSC, noIR; P<0.01 compared with WT-NSC, IR | 115 |
| Fig. 1 J | WT-NSC, noIR | Relative Intracellular ROS | 1 | 0.11 | P<0.01 compared with  PD-NSC, noIR; P<0.01 compared with WT-NSC, IR | 10^5^ |
|  | PD-NSC, noIR | Relative Intracellular ROS | 1.65 | 0.05 | P<0.01 compared with  WT-NSC, noIR; P<0.001 compared with PD-NSC, IR | 10^5^ |
|  | WT-NSC, IR | Relative Intracellular ROS | 2.27 | 0.16 | P<0.01 compared with  WT-NSC, noIR; P<0.001 compared with PD-NSC, IR | 10^5^ |
|  | PD-NSC, IR | Relative Intracellular ROS | 8.62 | 0.33 | P<0.001 compared with PD-NSC, noIR; P<0.001 compared with WT-NSC, IR | 10^5^ |
| Fig. 1L | WT-NSC, noIR | Percent of Ki67 positive | 0.37 | 0.02 | P<0.001 compared with WT-NSC, IR; P<0.05 compared with  PD-NSC, noIR | 86 |
|  | WT-NSC, IR | Percent of Ki67 positive | 0.17 | 0.01 | P<0.001 compared with  WT-NSC, noIR; P<0.05 compared with PD-NSC, IR | 60 |
|  | PD-NSC, noIR | Percent of Ki67 positive | 0.30 | 0.03 | P<0.05 compared with  WT-NSC, noIR; P<0.001 compared with PD-NSC, IR | 131 |
|  | PD-NSC, IR | Percent of Ki67 positive | 0.08 | 0.03 | P<0.001 compared with PD-NSC, noIR; P<0.05 compared with WT-NSC, IR | 91 |
| Fig. 1N | WT-NSC, noIR | Percent of γH2AX positive | 0.5 | 0.17 | P<0.01 compared with WT-NSC, IR; ns compared with  PD-NSC, noIR | 48 |
|  | WT-NSC, IR | Percent of γH2AX positive | 1.89 | 0.19 | P<0.01 compared with  WT-NSC, noIR; P<0.01 compared with PD-NSC, IR | 51 |
|  | PD-NSC, noIR | Percent of γH2AX positive | 0.53 | 0.32 | ns compared with  WT-NSC, noIR; P<0.01 compared with PD-NSC, IR | 63 |
|  | PD-NSC, IR | Percent of γH2AX positive | 5.17 | 0.76 | P<0.01 compared with PD-NSC, noIR; P<0.01 compared with WT-NSC, IR | 72 |
| Fig. 2D | PD-NSC, noIR | Percent of SA-β-gal positive | 28 | 1 | P<0.001 compared with PD-NSC, IR | 83 |
|  | PD-NSC, IR | Percent of SA-β-gal positive | 50 | 2 | P<0.001 compared with  PD-NSC, noIR; P<0.001 compared with PD-NSC, IR+RV; | 112 |
|  | PD-NSC, IR+RV | Percent of SA-β-gal positive | 34 | 4 | P<0.001 compared with PD-NSC, IR | 71 |
| Fig. 2F | PD-NSC, noIR | Percent of Ki67 positive | 31 | 2.1 | P<0.001 compared with PD-NSC, IR | 121 |
|  | PD-NSC, IR | Percent of Ki67 positive | 9 | 0.7 | P<0.001 compared with  PD-NSC, noIR; P<0.05 compared with PD-NSC, IR+RV; | 91 |
|  | PD-NSC, IR+RV | Percent of Ki67 positive | 12 | 1.4 | P<0.05 compared with PD-NSC, IR | 99 |
| Fig. 3B | WT-NSC, Scramble, noIR | Percent of SA-β-gal positive | 21 | 1.4 | P<0.01 compared with WT-NSC, Scramble, IR; P<0.01 compared with WT-NSC,  Sh-Sirt1, noIR | 171 |
|  | WT-NSC, Scramble, IR | Percent of SA-β-gal positive | 33 | 2.3 | P<0.01 compared with  WT-NSC, Scramble, noIR;  P<0.01 compared with WT-NSC,  Sh-Sirt1, IR | 135 |
|  | WT-NSC, Sh-Sirt1, noIR | Percent of SA-β-gal positive | 26 | 1 | P<0.01 compared with  WT-NSC, Scramble, noIR; P<0.001 compared with WT-NSC,  Sh-Sirt1, IR | 194 |
|  | WT-NSC, Sh-Sirt1, IR | Percent of SA-β-gal positive | 48 | 3 | P<0.01 compared with WT-NSC, Scramble, IR; P<0.001 compared with WT-NSC,  Sh-Sirt1, noIR | 150 |
| Fig. 3E | WT-NSC, Scramble, noIR | Percent of Ki67 positive | 34 | 2.1 | P<0.001 compared with WT-NSC, Scramble, IR; P<0.01 compared with WT-NSC,  Sh-Sirt1, noIR | 236 |
|  | WT-NSC, Scramble, IR | Percent of Ki67 positive | 11 | 1.1 | P<0.001 compared with  WT-NSC, Scramble, noIR;  P<0.001 compared with WT-NSC,  Sh-Sirt1, IR | 161 |
|  | WT-NSC, Sh-Sirt1, noIR | Percent of Ki67 positive | 21 | 2.5 | P<0.01 compared with  WT-NSC, Scramble, noIR; P<0.001 compared with WT-NSC,  Sh-Sirt1, IR | 341 |
|  | WT-NSC, Sh-Sirt1, IR | Percent of Ki67 positive | 1.4 | 0.2 | P<0.001 compared with WT-NSC, Scramble, IR; P<0.001 compared with WT-NSC,  Sh-Sirt1, noIR | 233 |
| Fig. 3G | WT-NSC, Scramble, noIR | Percent of γH2AX positive | 0.35 | 0.18 | P<0.001 compared with WT-NSC, Scramble, IR; ns compared with WT-NSC,  Sh-Sirt1, noIR | 60 |
|  | WT-NSC, Scramble, IR | Percent of γH2AX positive | 1.63 | 0.18 | P<0.001 compared with  WT-NSC, Scramble, noIR;  P<0.001 compared with WT-NSC,  Sh-Sirt1, IR | 74 |
|  | WT-NSC, Sh-Sirt1, noIR | Percent of γH2AX positive | 0.52 | 0.04 | ns compared with WT-NSC, Scramble, noIR; P<0.001 compared with WT-NSC,  Sh-Sirt1, IR | 75 |
|  | WT-NSC, Sh-Sirt1, IR | Percent of γH2AX positive | 4.15 | 0.4 | P<0.001 compared with WT-NSC, Scramble, IR; P<0.001 compared with WT-NSC,  Sh-Sirt1, noIR | 59 |
| Fig. 3I | PD-NSC, Control, noIR | Percent of Ki67 positive | 31 | 1.6 | P<0.001 compared with PD-NSC, Control, IR; P<0.01 compared with PD-NSC, Sirt1, noIR | 91 |
|  | PD-NSC, Control, IR | Percent of Ki67 positive | 4.4 | 1.4 | P<0.001 compared with  PD-NSC, Control, noIR; P<0.001 compared with PD-NSC, Sirt1, IR | 101 |
|  | PD-NSC, Sirt1, noIR | Percent of Ki67 positive | 45.8 | 2.6 | P<0.01 compared with  PD-NSC, Control, noIR; P<0.001 compared with PD-NSC, Sirt1, IR | 144 |
|  | PD-NSC, Sirt1, IR | Percent of Ki67 positive | 20.6 | 2.5 | P<0.001 compared with PD-NSC, Control, IR; P<0.001 compared with PD-NSC, Sirt1, noIR | 130 |
| Fig. 4B | PD-NSC, noIR | Percent of SA-β-gal positive | 18 | 2.1 | P<0.001 compared with PD-NSC, IR | 195 |
|  | PD-NSC, IR | Percent of SA-β-gal positive | 40 | 0.9 | P<0.001 compared with  PD-NSC, noIR; P<0.001 compared with PD-NSC, IR+SB; | 198 |
|  | PD-NSC, IR+SB | Percent of SA-β-gal positive | 24 | 3.1 | P<0.001 compared with PD-NSC, IR | 160 |
| Fig. 5E | WT-NSC, Con | Number of AVs per field | 3.8 | 1.5 | ns compared with WT-NSC, H_2_O_2_; ns compared with  PD-NSC, Con | 69 fields |
|  | WT-NSC, H_2_O_2_ | Number of AVs per field | 3.6 | 0.7 | ns compared with  WT-NSC, Con; P<0.001 compared with PD-NSC, H_2_O_2_ | 72 fields |
|  | PD-NSC, Con | Number of AVs per field | 1.9 | 0.4 | ns compared with  WT-NSC, Con; P<0.05 compared with PD-NSC, H_2_O_2_ | 82 fields |
|  | PD-NSC, H_2_O_2_ | Number of AVs per field | 1.3 | 0.4 | P<0.001 compared with WT-NSC, H_2_O_2_; P<0.05 compared with PD-NSC, Con | 108 fields |
| Fig. 5G | WT-NSC, Con | Autophagosomes (yellow dots) per cell | 8.8 | 1.3 | P<0.05 compared with WT-NSC, H_2_O_2_; P<0.01 compared with  PD-NSC, Con | 68 |
|  | WT-NSC, H_2_O_2_ | Autophagosomes (yellow dots) per cell | 5.7 | 0.6 | P<0.05 compared with  WT-NSC, Con; P<0.001 compared with PD-NSC, H_2_O_2_ | 60 |
|  | PD-NSC, Con | Autophagosomes (yellow dots) per cell | 3.5 | 0.4 | P<0.01 compared with  WT-NSC, Con; P<0.01 compared with PD-NSC, H_2_O_2_ | 80 |
|  | PD-NSC, H_2_O_2_ | Autophagosomes (yellow dots) per cell | 1.6 | 0.3 | P<0.001 compared with WT-NSC, H_2_O_2_; P<0.01 compared with PD-NSC, Con | 83 |
| Fig. 6B | PD-NSC, noIR | Percent of SA-β-gal positive | 21.4 | 2.5 | P<0.001 compared with PD-NSC, IR | 97 |
|  | PD-NSC, IR | Percent of SA-β-gal positive | 39.4 | 1.1 | P<0.001 compared with  PD-NSC, noIR; P<0.001 compared with PD-NSC, IR+RA; P<0.05 compared with PD-NSC, IR+CQ | 121 |
|  | PD-NSC, IR+RA | Percent of SA-β-gal positive | 21.6 | 1.5 | P<0.001 compared with  PD-NSC, IR | 90 |
|  | PD-NSC, IR+CQ | Percent of SA-β-gal positive | 51.6 | 4.2 | P<0.05 compared with  PD-NSC, IR | 129 |
| Fig. 6E | PD-NSC, noIR | Percent of Ki67 positive | 32.3 | 1.2 | P<0.001 compared with PD-NSC, IR | 123 |
|  | PD-NSC, IR | Percent of Ki67 positive | 8.7 | 2.5 | P<0.001 compared with  PD-NSC, noIR; P<0.0001 compared with PD-NSC, IR+RA; P<0.05 compared with PD-NSC, IR+CQ | 117 |
|  | PD-NSC, IR+RA | Percent of Ki67 positive | 23.9 | 1.2 | P<0.001 compared with  PD-NSC, IR | 110 |
|  | PD-NSC, IR+CQ | Percent of Ki67 positive | 2.6 | 0.5 | P<0.05 compared with  PD-NSC, IR | 97 |
| Fig. 8B | PD-NSC, noIR | Percent of SA-β-gal positive | 21.8 | 1.7 | P<0.001 compared with PD-NSC, IR | 122 |
|  | PD-NSC, IR | Percent of SA-β-gal positive | 42.3 | 4.0 | P<0.001 compared with  PD-NSC, noIR; P<0.01 compared with PD-NSC, IR+RV; P<0.05 compared with PD-NSC, IR+CQ | 114 |
|  | PD-NSC, IR+RV | Percent of SA-β-gal positive | 25.9 | 1.0 | P<0.01 compared with  PD-NSC, IR; P<0.01 compared with PD-NSC, IR+RV+CQ | 112 |
|  | PD-NSC, IR+RV+CQ | Percent of SA-β-gal positive | 36.6 | 2.5 | P<0.01 compared with  PD-NSC, IR+RV; P<0.01 compared with PD-NSC, IR+CQ | 111 |
|  | PD-NSC, IR+CQ | Percent of SA-β-gal positive | 49.8 | 1.3 | P<0.05 compared with  PD-NSC, IR; P<0.01 compared with PD-NSC, IR+RV+CQ | 139 |
| Fig. 8E | siControl, Con | Percent of SA-β-gal positive | 28.0 | 1.8 | P<0.001 compared with siControl, H_2_O_2_; P<0.05 compared with  siBECN1, Con | 206 |
|  | siControl, H_2_O_2_ | Percent of SA-β-gal positive | 55.6 | 4.9 | P<0.001 compared with  siControl, Con; P<0.01 compared with siControl, H_2_O_2_+RV P<0.01 compared with siBECN1, H_2_O_2_ | 189 |
|  | siControl, H_2_O_2_+RV | Percent of SA-β-gal positive | 34.1 | 4.6 | P<0.01 compared with  siControl, H_2_O_2_; P<0.001 compared with siBECN1, H_2_O_2_+RV | 153 |
|  | siBECN1, Con | Percent of SA-β-gal positive | 36.0 | 2.9 | P<0.05 compared with siControl, Con; P<0.001 compared with siBECN1, H_2_O_2_ | 145 |
|  | siBECN1, H_2_O_2_ | Percent of SA-β-gal positive | 73.0 | 3.4 | P<0.01 compared with siControl, H_2_O_2_; P<0.001 compared with siBECN1, Con; P<0.05 compared with siBECN1, H_2_O_2_+RV | 234 |
|  | siBECN1, H_2_O_2_+RV | Percent of SA-β-gal positive | 63.3 | 2.8 | P<0.001 compared with siControl, H_2_O_2_+RV; P<0.05 compared with siBECN1, H_2_O_2_ | 206 |
| Fig. S4 | WT-NSC, no H_2_O_2_ | Percentage of SA-b-gal positive cells | 0.24 | 0.55 | ns compared with WT-NSC, H_2_O_2_; ns compared with  PD-S-NSC, no H_2_O_2_ | 193 |
|  | WT-NSC, H_2_O_2_ | Percentage of SA-b-gal positive cells | 0.30 | 0.02 | ns compared with  WT-NSC, no H_2_O_2_; P<0.001 compared with PD-S-NSC, H_2_O_2_ | 221 |
|  | PD-S-NSC, no H_2_O_2_ | Percentage of SA-b-gal positive cells | 0.33 | 0.05 | ns compared with  WT-NSC, no H_2_O_2_; P<0.01 compared with PD-S-NSC, H_2_O_2_ | 191 |
|  | PD-S-NSC, H_2_O_2_ | Percentage of SA-b-gal positive cells | 0.52 | 0.02 | P<0.001 compared with WT-NSC, H_2_O_2_; P<0.01 compared with PD-S-NSC, no H_2_O_2_ | 161 |
| Fig. S5 | PD-S-NSC, H_2_O_2_ | Percentage of SA-b-gal positive cells | 0.55 | 0.02 | P<0.001 compared with PD-S-NSC, H_2_O_2_+RV; P<0.001 compared with PD-S-NSC, H_2_O_2_+RA | 191 |
|  | PD-S-NSC, H_2_O_2_+RV | Percentage of SA-b-gal positive cells | 0.36 | 0.01 | P<0.001 compared with  PD-S-NSC, H_2_O_2_ | 258 |
|  | PD-S-NSC, H_2_O_2_+RA | Percentage of SA-b-gal positive cells | 0.32 | 0.03 | P<0.001 compared with  PD-S-NSC, H_2_O_2_ | 193 |
